# Ankylosing Spondylitis and the Risk of Lung Cancer: A Meta-Analysis and Mendelian Randomization

**DOI:** 10.3389/fgene.2022.861984

**Published:** 2022-07-15

**Authors:** Yiyuan Ao, Yaokai Wen, Yutian Li, Haoxin Peng, Xiangrong Wu, Zhufeng Wang, Yu Jiang, Yuechun Lin, Shuben Li

**Affiliations:** ^1^ Department of Thoracic Surgery and Oncology, State Key Laboratory of Respiratory Disease, National Clinical Research Center for Respiratory Disease, The First Affiliated Hospital of Guangzhou Medical University, Guangzhou Institute of Respiratory Health, Guangzhou, China; ^2^ Nanshan School, Guangzhou Medical University, Guangzhou, China; ^3^ School of Medicine, Tongji University, Shanghai, China; ^4^ Department of Medical Oncology, Shanghai Pulmonary Hospital, Tongji University Medical School Cancer Institute, Tongji University School of Medicine, Shanghai, China

**Keywords:** lung cancer, ankylosing spondylitis, causality, Mendelian randomization, meta-analysis

## Abstract

**Background:** It remains uncertain whether ankylosing spondylitis is associated with an increased risk of lung cancer.

**Methods:** We conducted a meta-analysis to comprehensively evaluate the correlation between ankylosing spondylitis and lung cancer based on existing literature. Eligible studies were identified by searching the PubMed, Web of Science, Embase, and Cochrane Library before 26 March 2021. Subgroup analyses based on regions were also carried out. To further explore their causality, a two-sample Mendelian randomization analysis was performed, with 25 ankylosing spondylitis-related single nucleotide polymorphisms derived from the largest sample genome-wide association study of ankylosing spondylitis (ebi-a-GCST005529, 22,647 individuals). The inverse variance-weighted method was applied to estimate the causality, and the pleiotropy was assessed utilizing the Mendelian randomization-Egger regression approach.

**Results:** The meta-analysis including seven studies, with a total of 39,186 individuals, suggested no significant association between ankylosing spondylitis and lung cancer (relative risk, 1.10; 95% confidence interval, 0.89–1.36; I2, 61.8%). After excluding one study leading to high heterogeneity, we found that ankylosing spondylitis was associated with a 19% increased risk of lung cancer (relative risk, 1.19; 95% confidence interval, 1.01–1.40; I2, 0.0%). Subgroup analyses suggested that ankylosing spondylitis was not associated with increased risks of lung cancer in neither European (relative risk, 1.05; 95% confidence interval, 0.80–1.39; I2, 0.0%) nor non-European (relative risk, 1.14; 95% confidence interval, 0.84–1.55; I2, 79.6%) patients. Nevertheless, the Mendelian randomization results indicated that genetically determined ankylosing spondylitis was causally correlated with a remarkably increased risk of lung cancer among European populations (odds ratio, 1.26; 95% confidence interval, 1.07–1.48). Subgroup analyses further elucidated that genetically determined ankylosing spondylitis was causally associated with a notably higher risk of only squamous cell lung cancer (odds ratio, 1.39; 95% confidence interval, 1.05–1.83), rather than lung adenocarcinoma (odds ratio, 1.18; 95% confidence interval, 0.91–1.54). In addition, the results indicated the absence of pleiotropy.

**Conclusion:** The results of both modified meta-analysis and Mendelian randomization analysis suggested that ankylosing spondylitis was likely to be correlated with the development of lung cancer. Further research is warranted to clarify the specific mechanism regarding the causality between the two diseases.

## 1 Introduction

Inflammatory arthritis includes a heterogeneous group of chronic diseases. Although they are clinically and genetically distinct, symptoms are commonly redness, swelling, heat, pain, dysfunction, and deformities of the joints (Fang et al., 2020). Ankylosing spondylitis (AS) is the typical type of inflammatory arthritis, which usually affects the axial skeleton while presenting as low back pain along with morning stiffness, particularly among young males below 48 years of age (Braun and Sieper, 2007). Lung cancer is a leading cause of cancer death in the world (Nasim et al., 2019). Previous studies have reported that interstitial lung disease (ILD) was a pulmonary involvement in AS (Casserly et al., 1997; Souza et al., 2004; Baser et al., 2006). There is abundant evidence in epidemiological studies that ILD would increase the risk of lung cancer (Bouros et al., 2002; Archontogeorgis et al., 2012; Naccache et al., 2018). Previous studies showed that AS is associated with an increase in the overall risk for malignancy. (Deng et al., 2016; Chang et al., 2017; Nam et al., 2019).Thus, it is possible that AS could be a risk factor for lung cancer, theoretically. In addition, more frequent X-ray examinations among AS patients and inflammation resulting from AS might also play a role in the carcinogenic process (Picano et al., 2014). Therefore, whether the progress of AS will promote the occurrence of LC has received increasing attention.

Previous evidence regarding the association between AS and lung cancer is not always consistent. One cohort study including 4,133 AS patients and 16,532 controls illustrated that AS could increase the risk of lung cancer (Sun et al., 2014), while some other observational studies showed an absence of significantly higher incidence of lung cancer in AS population (Feltelius et al., 2003; Carmona et al., 2011; Hellgren et al., 2016; Chang et al., 2017; Kelty et al., 2021), some of which even found that AS played a role in the prevention of lung cancer (Ward and Alehashemi, 2020). Consequently, to comprehensively evaluate their correlation, we performed a meta-analysis established on current literature. Nonetheless, the limitations of conventional epidemiological studies still cannot be neglected, including different criteria for enrollment, small sample size, and the impacts of both potential confounders and inverse causality. Thus, generally, it is challenging to draw a reliable conclusion of causality based upon traditional epidemiological methods.

Mendelian randomization (MR) is an epidemiological study design, which can assess the causality between exposures and outcomes utilizing single nucleotide polymorphisms (SNPs) as genetic variants. Because genetic variants are typically associated with specific traits except for confounders and they are randomly allocated and fixed before birth, they could be considered unconfounded proxies for modifiable risk factors (Zhou et al., 2019). Differences in exposure to risk factors due to whether individuals carry genetic variants or not can lead to differences in outcomes (Emdin et al., 2017). Because genetic variants only affect the outcome by the variation in exposure, reverse causation can be circumvented. Differences in individuals carrying genetic variants in Mendelian analyses can be served as differences in interventions in randomized controlled trials (RCTs) (Davey Smith and Hemani, 2014). Confounding traits are equal between groups because of the random allocation of their genetic variants, which is also similar to RCTs. Based on the controversy of previous observational studies over the relation between AS and lung cancer (Feltelius et al., 2003; Carmona et al., 2011; Sun et al., 2014; Hellgren et al., 2016; Chang et al., 2017; Ward and Alehashemi, 2020; Kelty et al., 2021), we decided to utilize AS-related genetic variants as instrumental variables to further verify the results of our meta-analysis that whether AS could increase the risk of lung cancer. To our knowledge, it is the first MR analysis to evaluate the causality between AS and lung cancer risk. We present the following article in accordance with the Prisma reporting checklist.

## 2 Methods

### 2.1 Meta-Analysis

#### 2.1.1 Search Strategy

Eligible studies were identified by searching the PubMed, Web of Science, Embase, and Cochrane Library, with the following keywords: ankylosing spondylitis, lung cancer, and association as well as their Medical Subject Headings terms. Search strings are detailed in Supplementary Table S1. Additionally, a manual search of the list of references in review articles and conference abstracts was conducted. The cut-off date was 26 March 2021.

#### 2.1.2 Eligibility Criteria

The following criteria were adopted: 1) human participants without lung cancer history prior to being diagnosed as AS; 2) age range of patients and combined cancers other than lung cancer were not restricted; 3) studies as cohort designs; 4) lung cancer incidence data from certified registers; 5) studies investigating AS as the exposure and the incidence of lung cancer as the outcome; 6) studies providing one of the following indicators: risk ratio (RR), standardized incidence ratio (SIR), hazard ratio (HR), incidence rate ratio (IRR), or odds ratio (OR), to estimate lung cancer risk among AS patients, and 7) studies published in English.

#### 2.1.3 Study Selection and Data Extraction

Two authors (YYA and YTL) identified the eligible studies independently, with divergence solved by reaching a consensus after discussion with a senior investigator (SBL). We further screened the titles, abstracts, and full texts of all articles identified by our search to check the relevance and general adherence to eligibility criteria. The following data were extracted from included studies: the first author’s name, year of publication, country/region, study type, period of follow-up, sources of AS patients, adjusted variables, number of AS patients, number of lung cancer patients, and estimates of lung cancer risk among AS patients.

#### 2.1.4 Assessment of Research Quality

The quality of the studies was determined through Newcastle–Ottawa scale (NOS), which comprised the selection of the exposed and unexposed cohort, the comparability of the two cohorts, and the outcome assessment (Stang, 2010). The maximum score of NOS is 9 stars, and it is considered that a study of ≤3 stars as low quality, 4–6 stars as moderate quality, and ≥7 stars as high quality.

#### 2.1.5 Statistical Analysis

Due to the expected low risk of lung cancer overall, we could universally neglect the differences among the measures of RR, OR, SIR, and HR (Greenland, 1987). Therefore, to combine and calculate the data, we transformed them into RR values with corresponding 95% confidence intervals (CIs). I^2^-statistic was used to appraise the heterogeneity. The fixed-effects model was applied when low heterogeneity was observed (I^2^ < 25%); otherwise, the random-effects model was utilized. Stratified analyses based on the European region and non-European region were also conducted. Sensitivity analyses were performed by excluding one study every time and recalculating the combined estimates of the remaining studies to confirm whether the results could be markedly influenced by a single study. We conducted seven one-by-one elimination experiments in total. STATA 15.0 software was used to conduct the above statistical analyses.

### 2.2 MR Analysis

#### 2.2.1 Genetic Variants Associated With AS

The information on genetic variants relevant to AS was attained from the largest sample genome-wide association study (GWAS) of AS (dataset ID: ebi-a-GCST005529), with a total of 22,647 individuals (including 9,069 AS cases and 13,578 controls) (Cortes et al., 2013). It provided 26 loci under the selection criteria at the genome-wide significance threshold of *p* < 5 × 10^−8^, and none of them were excluded for surpassing the limited value (*r* ^2^ < 0.001) in linkage disequilibrium (LD) analysis. The following SNP (rs130075) missed the required information for the MR tests and was excluded. Finally, we used the remaining 25 genetic variants to estimate the causal effect of AS on the risk of lung cancer (Supplementary Table S2). These SNPs explained 24.4% of the variation in AS across individuals of European ancestry (Cortes et al., 2013).

#### 2.2.2 GWAS Summary Data on Lung Cancer

We retrieved GWAS summary data on lung cancer patients of European ancestry from the International Lung Cancer Consortium (ILCCO) (dataset ID: ieu-a-965, ieu-a-966, and ieu-a-967) (Wang et al., 2014), UK Biobank (dataset ID: ieu-b-4954), and Neale Lab (dataset ID: ukb-a-54), including 20,906 cases and 754,778 controls (Supplementary Table S3). For each of the 25 SNPs associated with AS, we retrieved summary data (the effects of each of the SNPs on the lung cancer; effect sizes and standard errors) from ILCCO (dataset ID: ieu-a-965, ieu-a-966, and ieu-a-967), UK Biobank (dataset ID: ieu-b-4954), and Neale Lab (dataset ID: ukb-a-54).

#### 2.2.3 Statistical Analysis

Power calculations were performed based on a method suggested by Burgess et al. (2014) to confirm whether the instrumental variables we use provide relatively accurate estimates of causal effects.

MR analyses were carried out through inverse-variance-weighted (IVW) method, which is the best unbiased estimate in the absence of pleiotropy (Bowden et al., 2015). This method consists of meta-analysis of the Wald ratio of each SNP between the exposure and the effect outcome to estimate the causal effect of AS on lung cancer. In addition, we also estimated the effects using the weighted median and the MR-Egger methods. The software used to analyze the data was R version (3.6.2) with the package “TwoSampleMR.” To get further comprehension of the causality between the AS and lung cancer, subgroup analyses according to histological subtypes of lung cancer (including lung adenocarcinoma and squamous cell lung carcinoma) were additionally conducted. The results were presented as ORs and corresponding 95% CIs.

#### 2.2.4 Sensitivity and Pleiotropy Analysis

Our MR analysis was based on three assumptions: 1) the SNPs are markedly relevant to AS; 2) the SNPs of AS are not associated with confounders; and 3) the AS-relevant SNPs are not directly related to lung cancer, and their effect on lung cancer can only be demonstrated by AS (Emdin et al., 2017). The first assumption was fulfilled since the included SNPs were selected at the genome-wide significance threshold of *p* < 5 × 10^−8^. Leave-one-out analysis was conducted by dropping out one SNP at a time and recalculating the IVW outcomes of the remaining SNPs, to testify whether the results of MR analysis could be biased or determined by a single SNP. We performed a Cochran’s Q test, which allows testing the differences across each SNP to test the existence of heterogeneity. The weighted median and MR-Egger regression approaches were performed to verify the second assumption. MR-Egger regression was conducted to test whether the selected SNPs exerted pleiotropy by accessing the intercept and its *p* value. We calculated the weighted median estimate, which can provide more precise estimates than those from MR-Egger regression if all genetic variants have similar magnitudes of association with the exposure (Bowden et al., 2016). The MR Pleiotropy Residual Sum and Outlier (PRESSO) test was conducted to identify and remove the outliers in order to eliminate the horizontal pleiotropy. For the third assumption, once these selected genetic variants are correlated with the development of lung cancer independent of AS, our MR analysis would not provide an accurate estimate of the causal impact of AS on lung cancer. So, we manually searched whether AS-related SNPs have secondary phenotypes other than AS in all the published GWASs. The cut-off date was 30 March 2021. Furthermore, according to previous epidemiological studies, smoking is an important environmental risk factor leading to both AS (Hwang et al., 2021) and lung cancer (Malhotra et al., 2016), thus it could be regarded as a confounder that can affect the causal relationship between AS and lung cancer. It was also reported that high intake of meat (particularly fried or well-done red meat), high alcohol consumption, and chronic obstructive pulmonary disease (COPD) were the risk factors for lung cancer (Malhotra et al., 2016), which could be considered as potential mediators of the AS–lung cancer relation. To testify whether smoking could (ever smoked and current tobacco smoking) affect the causal relationship between AS and lung cancer and investigate potential mechanisms from AS to lung cancer, additional MR analyses were conducted based upon six subsets from Medical Research Council Integrative Epidemiology Unit (MRC-IEU), Neale Lab, and UK Biobank (Supplementary Table S4).

## 3 Results

### 3.1 Meta-Analysis

#### 3.1.1 Characteristics and Quality Assessment of the Included Studies

**FIGURE 1:**
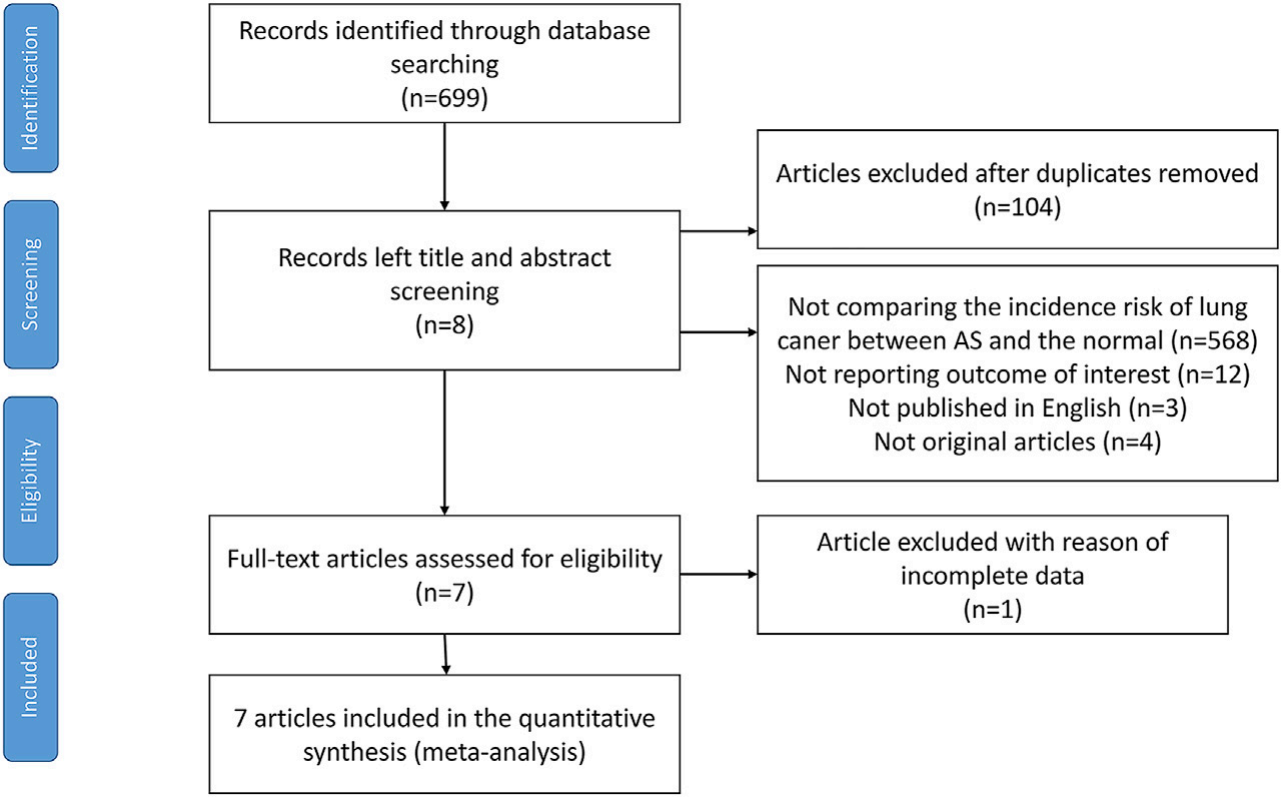
Flow chart containing the search strategies and identification of studies used in meta-analysis.

Electronic and manual searches yielded 699 potentially eligible articles with the mentioned search strategies. A flow chart of the screening of articles for inclusion in our meta-analysis is shown in Figure 1. During the first screening of titles and abstracts, 104 duplicate articles were excluded. Thereafter, 588 of the remaining 595 articles were excluded since 568 studies did not compare the incidence risk of lung cancer between AS patients and the general population, 12 studies did not report the target outcomes, 3 studies were not published in English, 4 studies did not provide full texts, and 1 study provided incomplete data. After careful examination, seven population-based cohort studies, which included 39,186 AS patients, met our criteria and were included in the meta-analysis (Feltelius et al., 2003; Carmona et al., 2011; Sun et al., 2014; Hellgren et al., 2016; Chang et al., 2017; Ward and Alehashemi, 2020; Kelty et al., 2021). Among them, three were from Europe, two from Asia, one from America, and one from Australia. The characteristics of the selected studies are provided in Table 1. Their quality scores ranged from 5 to 7 stars, with 6 of 7 studies scoring 7 stars (high quality) (Supplementary Table S5).

**TABLE 1:** Characteristics of the included studies in the meta-analysis.

Study	Region	Sources of AS patients	Design	Study period	Age range (year)	Adjusted variables	No. of AS patients (sex)	AS patients with lung cancer	Type of measure	RR (95%CI)
Carmona et al. (2011)	Spain	BIOBADASER 2.0 Cohort	Cohort	1999–2005	NA	Age, sex	761 (NA)	NA	SIR	1.66 (0.34, 4.85)
Chang et al. (2017)	China	Taiwan National Health Insurance Research Database	Cohort	2000–2008	≥ 16	NA	5,452 (2,913 males and 2,539 females)	44	SIR	1.07 (0.79, 1.43)
Feltelius et al. (2003)	Sweden	Swedish Hospital Inpatient Register	Cohort	1965–1995	≥ 0	Sex, age at entry, attained age at cancer diagnosis, follow-up years	6,621 (NA)	34	SIR	1.05 (0.73, 1.47)
Hellgren et al. (2016)	Sweden	Swedish National Patient and Population Registers	Cohort	2001–2011	≥ 16	Age, sex	7,023 (NA)	21	RR	1.0 (0.6, 1.6)
Kelty et al. (2021)	Australia	Western Australia Rheumatic Disease Epidemiological Register	Cohort	1980–2014	≥18	NA (number of cases too small for multivariable analysis)	2,152 (1,294 males and 858 females)	33	HR	1.33 (0.91, 1.95)
Sun et al. (2014)	China	National Health Insurance system of Taiwan	Cohort	1995–2010	≥ 0	Age, gender, hypertension, hyperlipidemia, diabetes, ischemic heart disease, asthma, COPD	4,133 (2,215 males and 1918 females)	36	HR	1.63 (1.10, 2.40)
Ward et al. (2020)	America	US Medicare fee-for-service hospitalization and outpatient data	Cohort	1999–2015	≥ 65	Age, sex, race, socioeconomic characteristics, geographic region, smoking, presence of COPD	13,044 (8,609 males and 4,435 females)	274	IRR	0.84 (0.74, 0.95)

COPD, chronic obstructive pulmonary disease; NA, not accessible; AS, ankylosing spondylitis; IRR, incidence rate ratio; HR, hazard ratio; RR, relative risk; SIR, standardized incidence ratio; CI, confidence interval.

#### 3.1.2 Risk of Lung Cancer Among AS Patients

**FIGURE 2:**
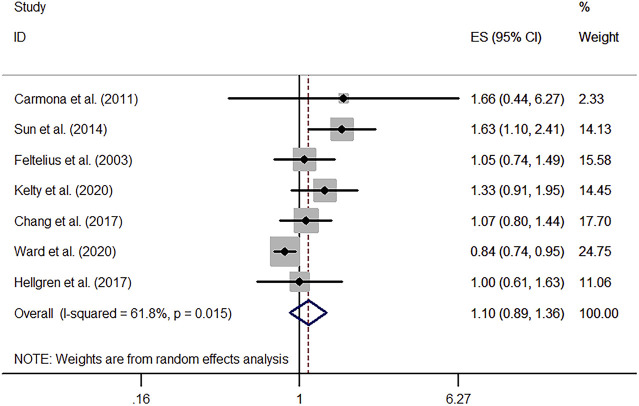
Forest plot of lung cancer risk among patients with ankylosing spondylitis.

Our meta-analysis demonstrated that AS was associated with an increased risk of lung cancer (RR, 1.10; 95% CI, 0.89–1.36) while without statistical significance. However, a high heterogeneity was observed (I^2^, 61.8%; *p* < 0.05). The corresponding forest plot is shown in Figure 2. Furthermore, subgroup analyses based on regions demonstrated no remarkable correlation between AS and lung cancer in both European (RR, 1.05; 95% CI, 0.80–1.39) (I^2^, 0.0%; *p* = 0.781) and non-European regions (RR, 1.14; 95% CI, 0.84–1.55) (I^2^, 79.6%; *p* < 0.05) (Supplementary Figure S1).

#### 3.1.3 Sensitivity Analysis

**FIGURE 3:**
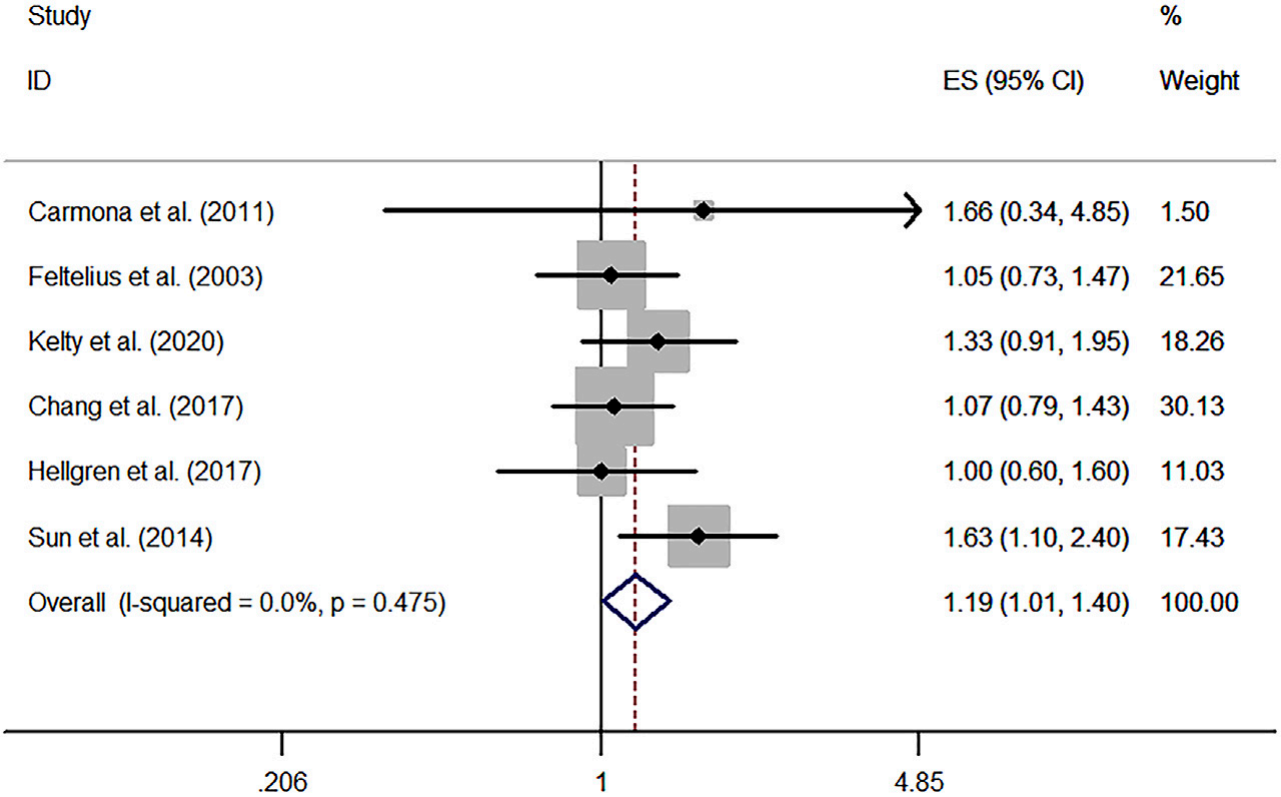
Modified forest plot of lung cancer risk among patients with ankylosing spondylitis.

In order to find the source of heterogeneity, sensitivity analysis was carried out by dropping out one study at a time and recalculating the combined estimates of the remaining studies to testify whether the results could be affected significantly by a single study. When excluding the study of Ward and Alehashemi (2020), a notable fluctuation was observed for the result that AS was associated with a 19% increased risk of lung cancer, with a low heterogeneity (RR, 1.19; 95% CI, 1.01–1.40; I^2^, 0.0%; *p* = 0.475) (Figure 3). The initial outcomes did not vary markedly when removing other studies (Supplementary Figure S2).

### 3.2 MR Analysis

#### 3.2.1 Power Calculation

We fixed the type I error rate at 0.05. Under the current sample size (11,348 cases of lung cancer and 15,861 controls) and the supposition of the 25 selected SNPs explaining a total of 24.4% variance of AS, our study had an estimated 100.0% power to detect the estimated causal effect size of AS (RR = 0.84) (Ward and Alehashemi, 2020) reported previously.

#### 3.2.2 MR Results of the Causality Between AS and Lung Cancer

Estimation of Wald ratio between each SNP related to AS and risk of lung cancer and its subtypes are shown in Supplementary Table S6. Our results showed genetically predicted AS was causally correlated with a markedly increased lung cancer (dataset ID: ieu-a-966) risk among European populations (OR, 1.26; 95% CI, 1.07–1.48; *p* = 0.005). However, we found that the findings were inconsistent when utilizing two new lung cancer datasets (dataset ID: ieu-b-4954; OR, 1.0009; 95% CI, 0.9987–1.0031; *p* = 0.4395) (dataset ID: ukb-a-54; OR, 1.0002; 95% CI, 0.9996–1.0007; *p* = 0.5234), showing that AS was not casually associated with lung cancer. Subgroup analyses further indicated that AS patients had a significantly higher risk of only squamous cell lung cancer (dataset ID: ieu-a-967; OR, 1.39; 95% CI, 1.05–1.83; *p* = 0.02), while null casual correlation was observed between AS and lung adenocarcinoma (dataset ID: ieu-a-965; OR, 1.18; 95% CI, 0.91–1.54; *p* = 0.21). We observed similar associations using the weighted-median and MR-Egger method (Table 2) (Supplementary Figures S3–S7).

**TABLE 2:** The estimates of the causality between AS and lung cancer in Mendelian randomization.

Outcome	IVW method		MR-Egger		Weighted median method	
	Or (95%CI)	*p*-value	Or (95%CI)	*p*-value	Or (95%CI)	*p*-value
Lung cancer overall (id: ieu-a-966)	1.26 (1.07, 1.48)	0.0045	1.41 (1.08, 1.83)	0.0191	1.32 (1.08, 1.61)	0.0073
Adenocarcinoma (id: ieu-a-965)	1.18 (0.91,1.54)	0.2141	1.35 (0.86, 2.10)	0.2039	1.26 (0.91, 1.74)	0.1646
Squamous cell carcinoma (id: ieu-a-967)	1.39 (1.05,1.83)	0.0210	1.48 (0.93, 2.35)	0.1152	1.42 (1.04, 1.93)	0.0263
Lung cancer (id: ieu-b-4954)	1.0009 (0.9987,1.0031)	0.4395	0.9997 (0.9960, 1.0035)	0.8952	1.0014 (0.9990, 1.0038)	0.2390
Cancer code self-reported: lung cancer (id: ukb-a-54)	1.0002 (0.9996,1.0007)	0.5234	1.0005 (0.9996, 1.0014	0.2676	1.0002 (0.9995, 1.0009)	0.6039

#### 3.2.3 Sensitivity Analysis

The MR-Egger regression showed no evidence to support the existence of unbalanced pleiotropy (intercept β = −0.0072, *p* = 0.3146 for lung cancer || id:ieu-a-966; intercept β = −0.0083, *p* = 0.4829 for lung adenocarcinoma || id:ieu-a-965; intercept β = −0.0042, *p* = 0.7377 for squamous cell lung cancer || id:ieu-a-967; intercept β = 0.0000718, *p* = 0.4751 for lung cancer || id:ieu-b-4954; and intercept β = −0.000021, *p* = 0.2320 for cancer code self-reported: lung cancer || id:ukb-a-54) (Supplementary Table S7). We did not find outlier SNPs or the horizontal pleiotropic effect using MR-PRESSO test (*p* = 0.402). In addition, we did not find any single SNP had significant impact on the overall effect of AS on lung cancer risk in terms of leave-one-out studies (Supplementary Figures S3–S7). We found no evidence that the included AS-associated SNPs have secondary phenotypes other than AS during our manual search. Furthermore, additional MR analyses were conducted to identify whether AS–lung cancer relation was impacted by the potential confounder (ever smoked and current tobacco smoking) or mediated by mediators (high intake of meat, high alcohol consumption, and COPD). We observed that genetically predisposed smoking was not significantly associated with AS (OR 1.7763, 95% CI 0.6432–4.9049, *p* = 0.2676 for ever smoked; OR 0.7538, 95% CI 0.4258–1.3344, *p* = 0.3321 for current tobacco smoking). The result also indicated that genetically predicted AS was not causally correlated with the potential mediators (OR 1.0032, 95% CI 0.9749–1.0322, *p* = 0.8285 for alcohol consumption; OR 1.0006, 95% CI 0.9993–1.0018, *p* = 0.3582 for COPD; OR 1.0073, 95% CI 0.9856–1.0295, *p* = 0.511 for meat consumers; OR 1.006, 95% CI 0.9840–1.0286, *p* = 0.5956 for processed meat intake). (Supplementary Tables S8, S9). According to the results of Cochran’s Q test, there was no heterogeneity among these SNPs (Supplementary Table S10).

## 4 Discussion

The original meta-analysis suggested no significant association between AS and lung cancer. The results of both modified meta-analysis and MR analysis suggested that AS was likely to be correlated with the development of lung cancer.

Compared with one previous study (Deng et al., 2016), our updated meta-analysis was more comprehensive for having a larger sample size. Seven published population-based cohort studies, including 39,186 individuals from numerous regions, were enrolled in our meta-analysis. The results suggested no significant correlation between AS and lung cancer. Nonetheless, a markedly high heterogeneity (I^2^, 61.8%; *p* < 0.05) was observed for this result. Subgroup analyses further illustrated that AS was not associated with increased risks of lung cancer in both European and non-European regions. To explore the source of heterogeneity, we investigated the influence of each single study on the overall risk estimate by excluding one study at a time. Noticeable change in the results of the remaining studies was observed after omitting the study of Ward and Alehashemi (2020), denoting that AS patients were associated with a 19% increase in the risk of lung cancer, with a low heterogeneity (I^2^, 0.0%; *p* = 0.475). We found that the study population of Ward’s study (Ward and Alehashemi, 2020) were those who had reached the age threshold of 65 years, while wide age ranges of study populations were observed in other included studies. Thus, the different characteristics of observational studies might contribute to the fluctuation of the results and the source of high heterogeneity. In addition, the differences among the adjusted variables of different studies could also lead to elevated heterogeneity (Table 1). Still, confounders without full elimination and rectification may influence the estimation of causality between AS and lung cancer in the conventional epidemiological settings. For example, some observational studies lacked control for smoking, which is considered a significant risk factor for both lung cancer and AS. With the impact of the potential confounders, the AS–lung cancer relation might not be evaluated that accurately.

Considering that the causality cannot be directly inferred from conventional epidemiological studies for their limitations, MR analysis may offer a new approach to assess the causality between exposures and diseases by using the genetic variants (Davey Smith and Ebrahim, 2003). These genetic variants are immutable because they are assigned to individuals before any exposure or outcome occurs, thus reducing the occurrence of reverse causation and potential confounders (Emdin et al., 2017). We obtained the information on genetic variants associated with AS and lung cancer from large sample GWASs. The causality between AS and different histological subtypes of lung cancer was also explored, which was difficult to achieve in previous observational studies. To our knowledge, this is the first MR analysis investigating the causation between AS and lung cancer. Our MR results indicated that genetically predicted AS was causally correlated with a notably increased risk of lung cancer (dataset ID: ieu-a-966), while such correlation was not observed in two other lung cancer datasets (dataset ID: ieu-b-4954 and ukb-a-54). For specific histological subtype, genetically predicted AS patients were significantly associated with a 39% increased risk of squamous cell lung carcinoma (dataset ID: ieu-a-967), while no significant causality was observed between them and lung adenocarcinoma (dataset ID: ieu-a-965). Meanwhile, our MR analysis had sufficient statistical power to provide a relatively accurate estimate of the causal effect, with a large sample size (*n* = 27,209) and robustly associated instrumental variables (F-statistics = 8782.74).

The MR analysis was valid only if three assumptions (Emdin et al., 2017) were satisfied. The first MR assumption was fulfilled since the selected included SNPs were selected at the genome-wide significance threshold of *p* < 5 × 10^−8^. The second MR assumption was also not likely to be violated since the results showed no statistical significance through the MR-Egger regression method and MR-PRESSO test, suggesting that the impact of pleiotropy could be negligible. In terms of the third MR assumption, 25 AS-associated SNPs were not genome-wide significantly associated with lung cancer in all published GWASs. Furthermore, we applied additional MR analyses, which demonstrated that the overall correlation between genetically predicted AS and the potential confounder (ever smoked and current tobacco smoking) and the mediators (high intake of meat, high alcohol consumption, and COPD) was not statistically significant, thus the third MR assumption was unlikely to be violated in our study either.

Mechanisms concerning the relevance between AS and lung cancer have been widely investigated, whereas none of them have been illustrated exactly. The most plausible explanation is that both inflammatory factors and genetic locus contribute to the relationship between lung cancer and AS. Human lymphocyte antigen-B27 (HLA-B27) is considered one of the factors related to cancer risk in AS (Feltelius et al., 2003). HLA-B27 has been proved to increase the risk of developing hematological malignancies among AS patients, and the plausible mechanism might be the reduced immune surveillance, molecular mimicry by oncogenic microbes, or putative linkage disequilibrium with yet unidentified susceptibility genes in the studied populations (Dorak et al., 1999; Au et al., 2001). An increased risk of the inflammatory disease occurrence was observed in individuals inheriting the human major histocompatibility complex class I allele HLA-B27, under the action of microbiome (Taurog et al., 1994; Rosenbaum and Asquith, 2018). The peptides presented by HLA-B27 are associated with endoplasmic reticulum aminopeptidase (Evans et al., 2011), which is involved in process of interleukin-23 (IL-23) receptor (Cortes et al., 2013), activating the inflammatory cells and the secretion of interleukin-17 (IL-17) (Sieper and Poddubnyy, 2017). More IL-17 would be produced by Killer-cell immunoglobulin-like receptor 3DL2(KIR3DL2) + cluster of differentiation 4(CD4) + T cells in the presence of IL-23 (Bowness et al., 2011). It was reported that IL-17-producing KIR3DL2 + CD4 + T cells would produce tumor necrosis factor-α (TNF-α) (Chen et al., 2017). The production of TNF-α by inflammatory cells can facilitate the survival of tumor cells (Luo et al., 2004; Lin and Karin, 2007). In addition, increased IL-17 level was detected in primary non-small cell lung cancer tissues of animal models, which is associated with increased tumor vascularity (Numasaki et al., 2005). The results of our subgroup MR analysis suggested that genetically determined AS was associated with an elevated risk of squamous cell lung carcinoma but not lung adenocarcinoma, thus further clinical and laboratory research are required to provide a more reliable conclusion for the causal effect and the relevant mechanisms between the two diseases.

Several limitations could not be neglected in our meta-analysis. First, the difference in age threshold of population and adjusted variables among included studies might be the cause of high heterogeneity. We excluded one study after identifying the source of heterogeneity, which might not only introduce bias but also reduce heterogeneity. Second, subgroup analyses were not able to be conducted in meta-analysis because of the limited original data from selected population-based cohort studies, therefore the effects of other confounding factors (age, gender, smoking, medication, and occupational factors) and the relation between AS and the subtypes of lung cancer could not be evaluated.

Limitations of MR analysis also existed. First, we only included 25 SNPs from one published GWAS. Second, due to the inconsistency of correlation between AS and lung cancer we observed in different datasets, further studies using more comprehensive GWAS are needed to obtain more clear results. Third, samples enrolled in our MR analysis were restricted to a population of European ancestry. Therefore, a larger sample study with more AS-related SNPs and more ethnicities might help provide a more valid conclusion for the causal relationship between AS and lung cancer. Fourth, several confounders of AS–lung cancer relationship still cannot be implemented by MR estimation separately (Burgess et al., 2015). Behavioral factors are difficult to be analyzed simply by MR analysis. To exemplify, medical radiation might increase the risk of lung cancer (Picano et al., 2014), a MR analysis based on AS patients with a long duration of medical radiation would be more powerful to demonstrate the causality with medicinal intervention. However, due to the finite data that we could acquire, we could not clarify whether medical radiation would affect the correlation between AS and lung cancer. Fifth, considering that some of the SNPs used in our study might be potentially correlated with some unknown factors that may be associated with lung cancer risk, the validity of the third hypothesis needed to be studied in more depth.

## 5 Conclusion

Overall, both the modified meta-analysis and MR analysis indicated an increased risk of lung cancer among AS patients. It is important for patients with AS to assess their physical status and control the modifiable risk factors of lung cancer, and the mechanism of the association between AS and lung cancer needs to be further investigated.

## Data Availability

The original contributions presented in the study are included in the article/Supplementary Material; further inquiries can be directed to the corresponding author.

## Abbreviations

AS, ankylosing spondylitis; CD4, cluster of differentiation 4; CI, confidence interval; COPD, chronic obstructive pulmonary disease; GWAS, genome-wide association study; HLA-B27, human lymphocyte antigen-B27; HR, hazard ratio; IL-17, interleukin-17; IL-23, interleukin-23; ILCCO, International Lung Cancer Consortium; ILD, Interstitial lung disease; IRR, incidence rate ratio; IVW, inverse-variance-weighted method; KIR3DL2, immunoglobulin-like receptor 3DL2; LD, linkage disequilibrium; MR, Mendelian randomization; MRC-IEU, Medical Research Council Integrative Epidemiology Unit; NOS, Newcastle–Ottawa scale; OR, odds ratio; PRESSO, Pleiotropy Residual Sum and Outlier; RCTs, randomized controlled trials; RR, relative risk; SIR, standardized incidence ratio; SNPs, single nucleotide polymorphisms; TNF-α, tumor necrosis factor-α.
