# Supplementary material for: Ankylosing Spondylitis and the Risk of Lung Cancer: A Meta-Analysis and Mendelian Randomization
Source: Front Genet. 2022 Jul 15;13:861984. doi: 10.3389/fgene.2022.861984 (PMC9337881; doi:10.3389/fgene.2022.861984)
Supplement: Supplementary file 1 [file DataSheet1.PDF]

Supplementary figures

Supplementary Figure 1. Forest plot of of lung cancer risk among patients with ankylosing spondylitis,
stratified by regions.

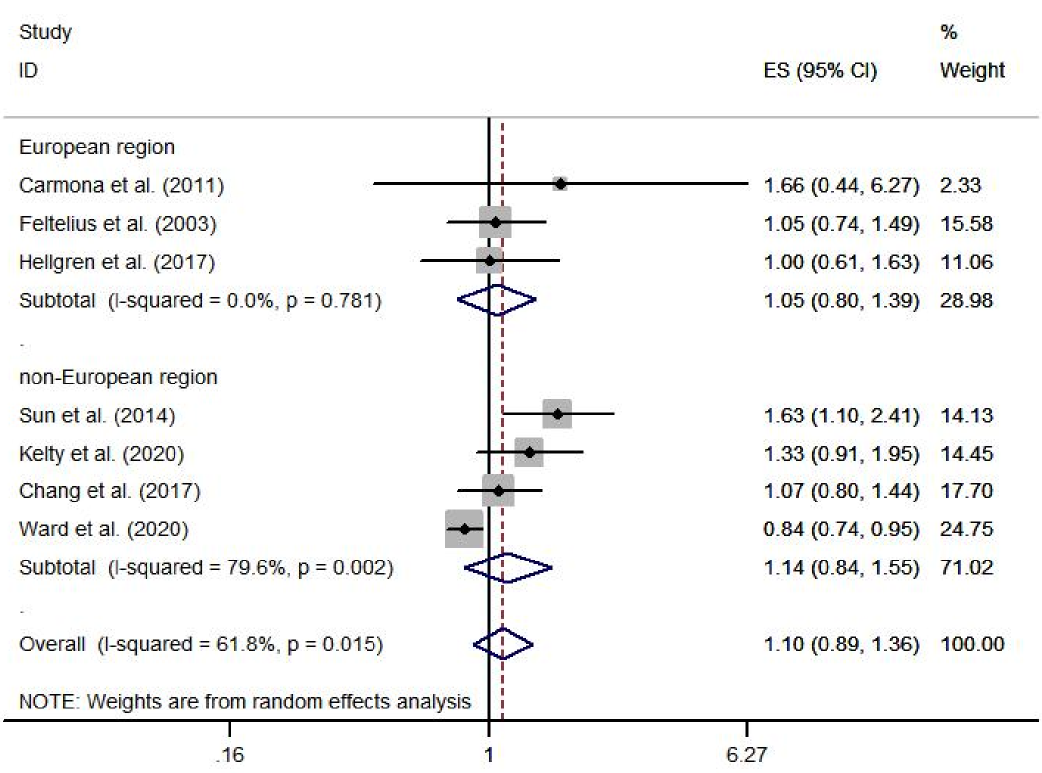

Supplementary Figure 2. Results of one-by-one elimination experiment

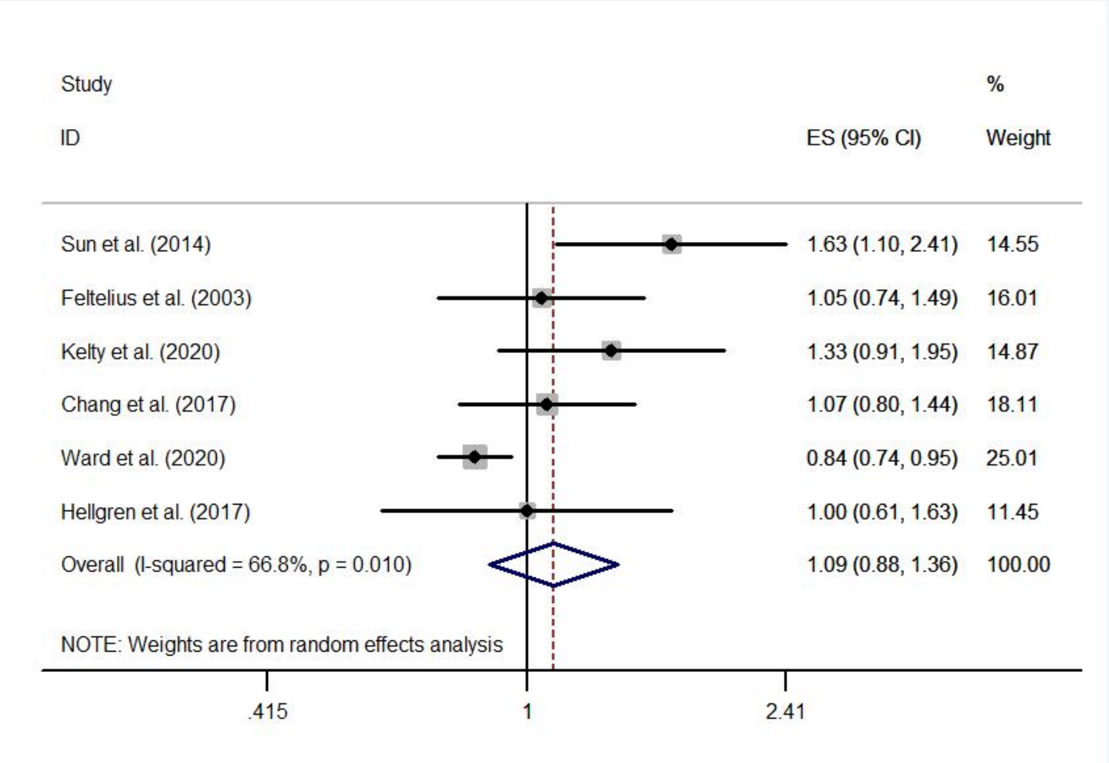
Study
ID
ES (95% CI)
%
Weight
Sun et al. (2014)
1.63 (1.10, 2.41)
14.55
Feltelius et al. (2003)
1.05 (0.74, 1.49)
16.01
Kelty et al. (2020)
1.33 (0.91, 1.95)
14.87
Chang et al. (2017)
1.07 (0.80, 1.44)
18.11
Ward et al. (2020)
0.84 (0.74, 0.95)
25.01
Hellgren et al. (2017)
1.00 (0.61, 1.63)
11.45
Overall (I-squared = 66.8%, p = 0.010)
1.09 (0.88, 1.36)
100.00
NOTE: Weights are from random effects analysis
.415
1
2.41

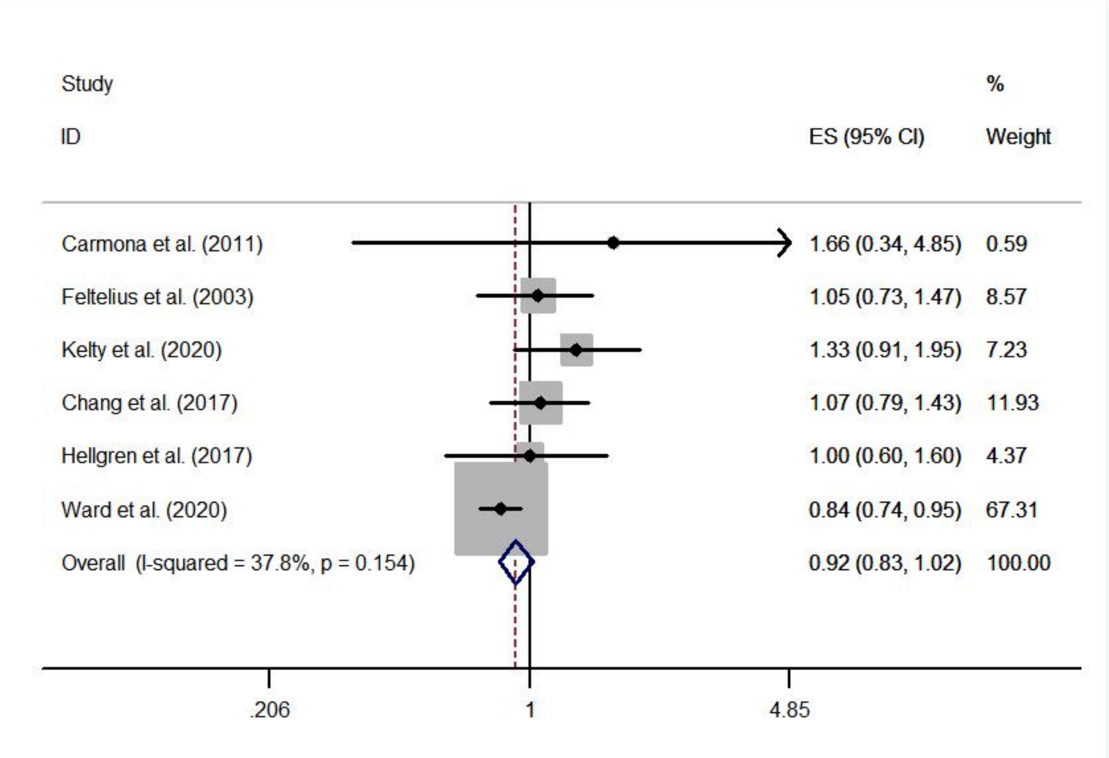
Study
ID
ES (95% CI)
%
Weight
Carmona et al. (2011)
1.66 (0.34, 4.85)
0.59
Feltelius et al. (2003)
1.05 (0.73, 1.47)
8.57
Kelty et al. (2020)
1.33 (0.91, 1.95)
7.23
Chang et al. (2017)
1.07 (0.79, 1.43)
11.93
Hellgren et al. (2017)
1.00 (0.60, 1.60)
4.37
Ward et al. (2020)
0.84 (0.74, 0.95)
67.31
Overall (I-squared = 37.8%, p = 0.154)
0.92 (0.83, 1.02)
100.00
.206
1
4.85

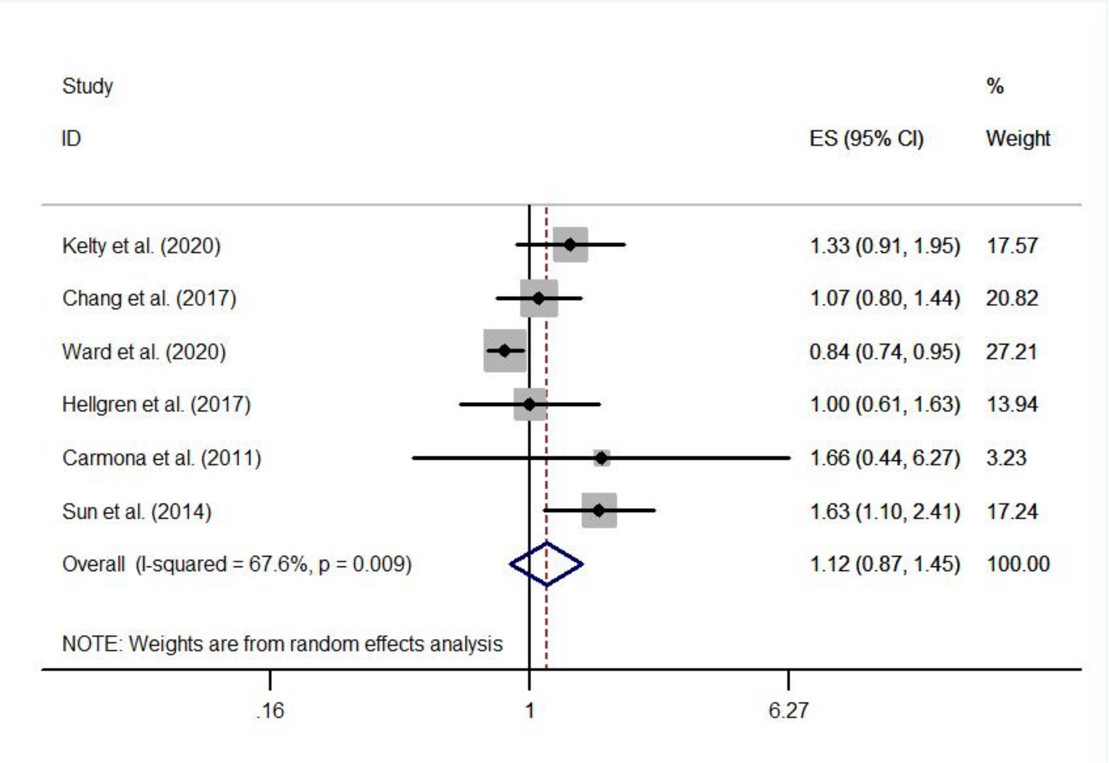
Study
ID
ES (95% CI)
%
Weight
Kelty et al. (2020)
1.33 (0.91, 1.95)
17.57
Chang et al. (2017)
1.07 (0.80, 1.44)
20.82
Ward et al. (2020)
0.84 (0.74, 0.95)
27.21
Hellgren et al. (2017)
1.00 (0.61, 1.63)
13.94
Carmona et al. (2011)
1.66 (0.44, 6.27)
3.23
Sun et al. (2014)
1.63 (1.10, 2.41)
17.24
Overall (I-squared = 67.6%, p = 0.009)
1.12 (0.87, 1.45)
100.00
NOTE: Weights are from random effects analysis
.16
1
6.27

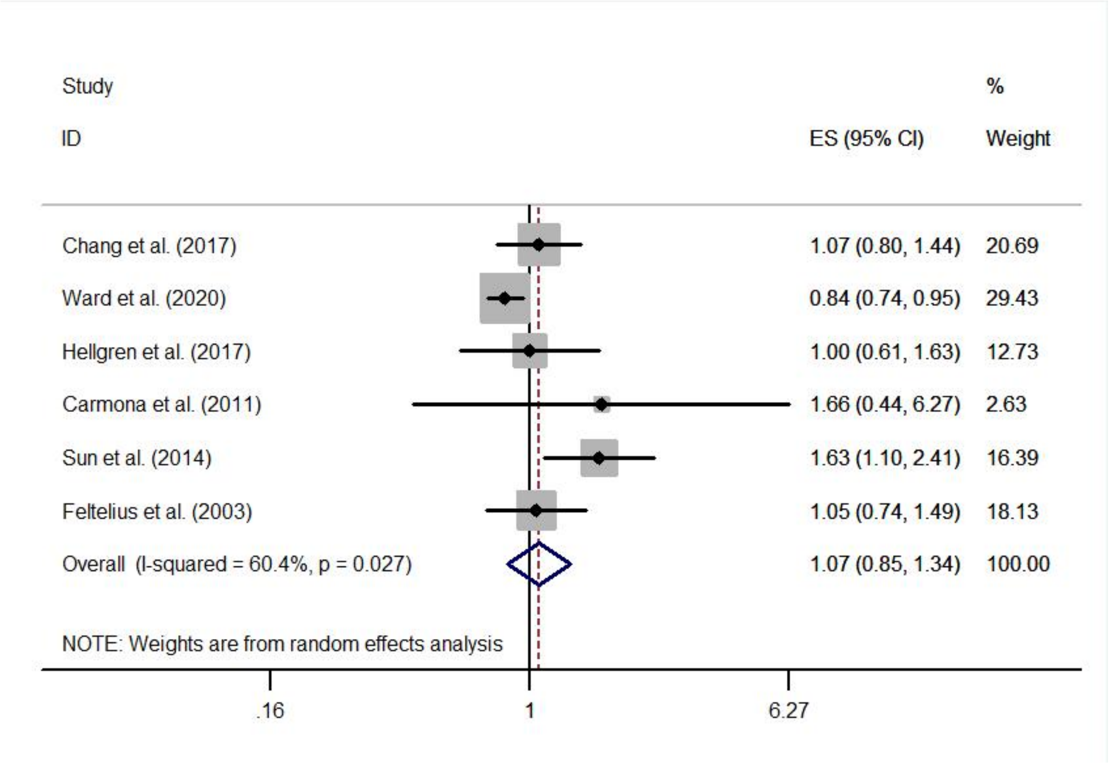
Study
ID
ES (95% CI)
%
Weight
Chang et al. (2017)
1.07 (0.80, 1.44)
20.69
Ward et al. (2020)
0.84 (0.74, 0.95)
29.43
Hellgren et al. (2017)
1.00 (0.61, 1.63)
12.73
Carmona et al. (2011)
1.66 (0.44, 6.27)
2.63
Sun et al. (2014)
1.63 (1.10, 2.41)
16.39
Feltelius et al. (2003)
1.05 (0.74, 1.49)
18.13
Overall (I-squared = 60.4%, p = 0.027)
1.07 (0.85, 1.34)
100.00
NOTE: Weights are from random effects analysis
.16
1
6.27

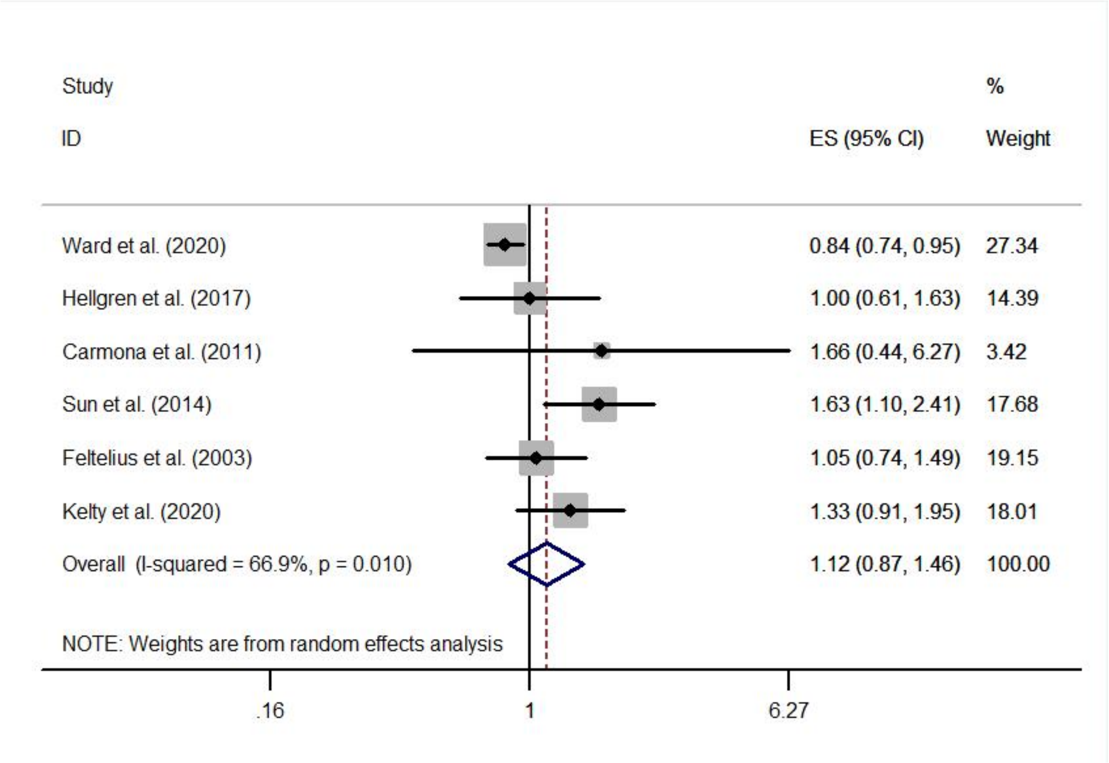
Study
ID
ES (95% CI)
%
Weight
Ward et al. (2020)
0.84 (0.74, 0.95)
27.34
Hellgren et al. (2017)
1.00 (0.61, 1.63)
14.39
Carmona et al. (2011)
1.66 (0.44, 6.27)
3.42
Sun et al. (2014)
1.63 (1.10, 2.41)
17.68
Feltelius et al. (2003)
1.05 (0.74, 1.49)
19.15
Kelty et al. (2020)
1.33 (0.91, 1.95)
18.01
Overall (I-squared = 66.9%, p = 0.010)
1.12 (0.87, 1.46)
100.00
NOTE: Weights are from random effects analysis
.16
1
6.27

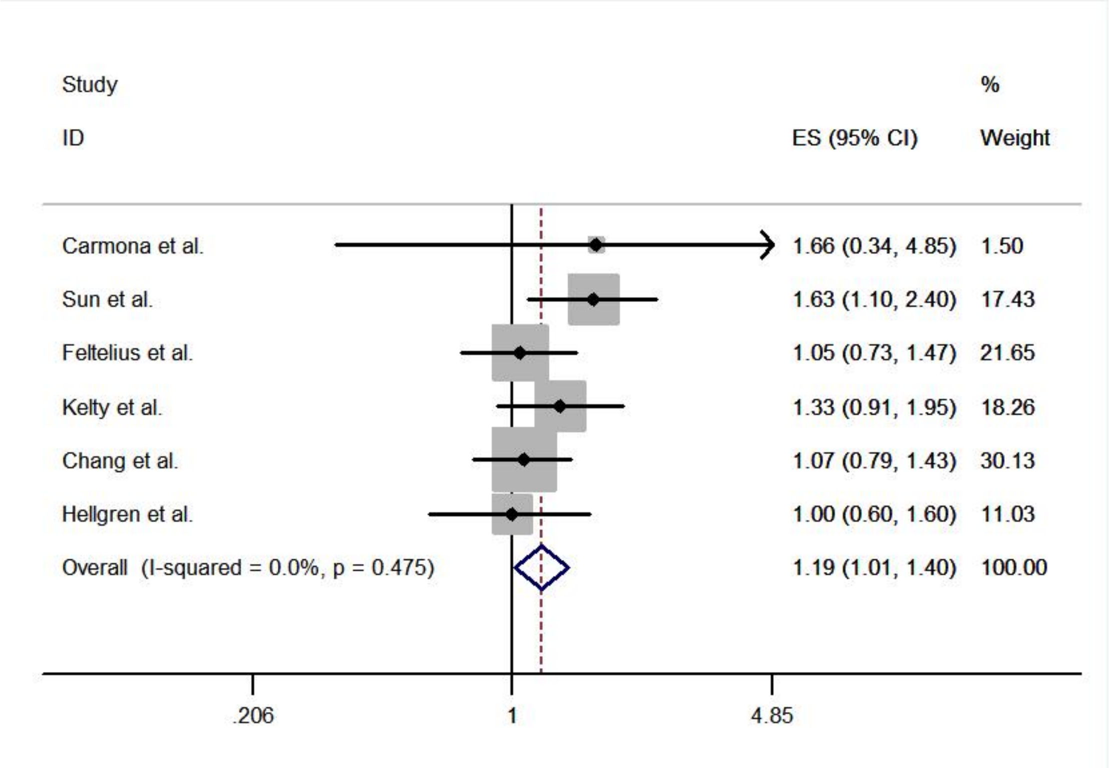
Study
ID
ES (95% CI)
%
Weight
Carmona et al.
1.66 (0.34, 4.85)
1.50
Sun et al.
1.63 (1.10, 2.40)
17.43
Feltelius et al.
1.05 (0.73, 1.47)
21.65
Kelty et al.
1.33 (0.91, 1.95)
18.26
Chang et al.
1.07 (0.79, 1.43)
30.13
Hellgren et al.
1.00 (0.60, 1.60)
11.03
Overall (I-squared = 0.0%, p = 0.475)
1.19 (1.01, 1.40)
100.00
.206
1
4.85

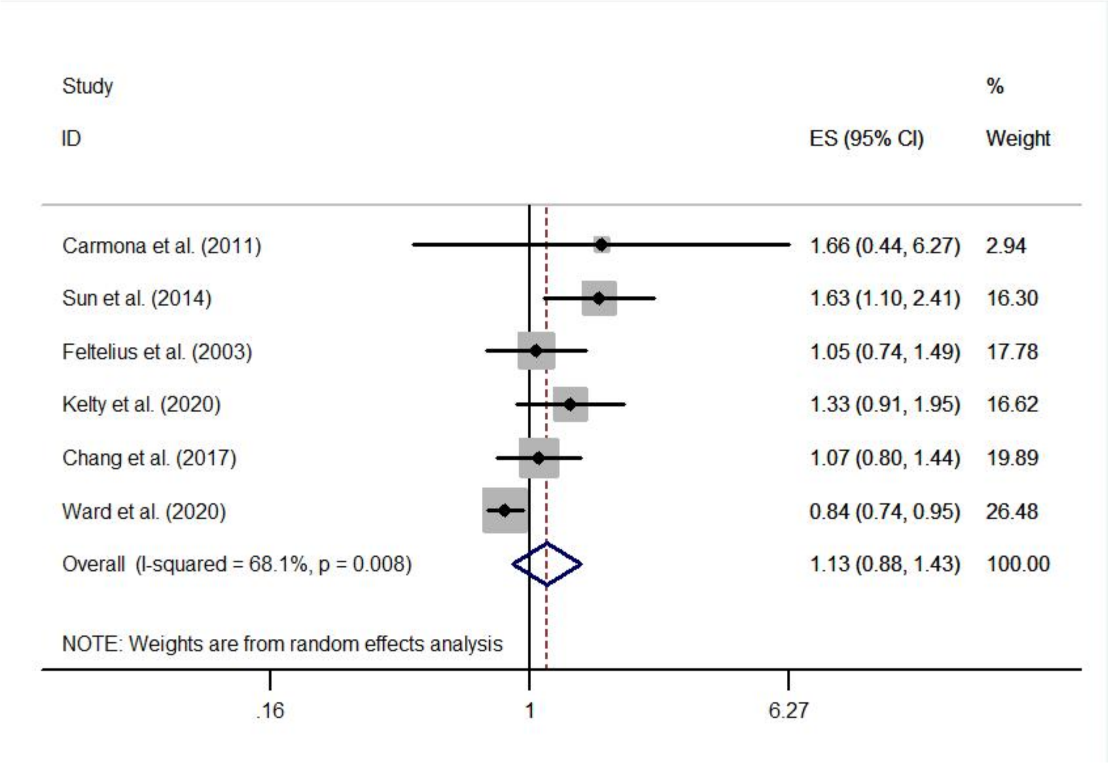

Supplementary Figure 3. MR effect size, leave-one-out analysis, MR-Egger scatter plot, and funnel plot for AS on lung adenocarcinoma (dataset ID: ieu-a-965)

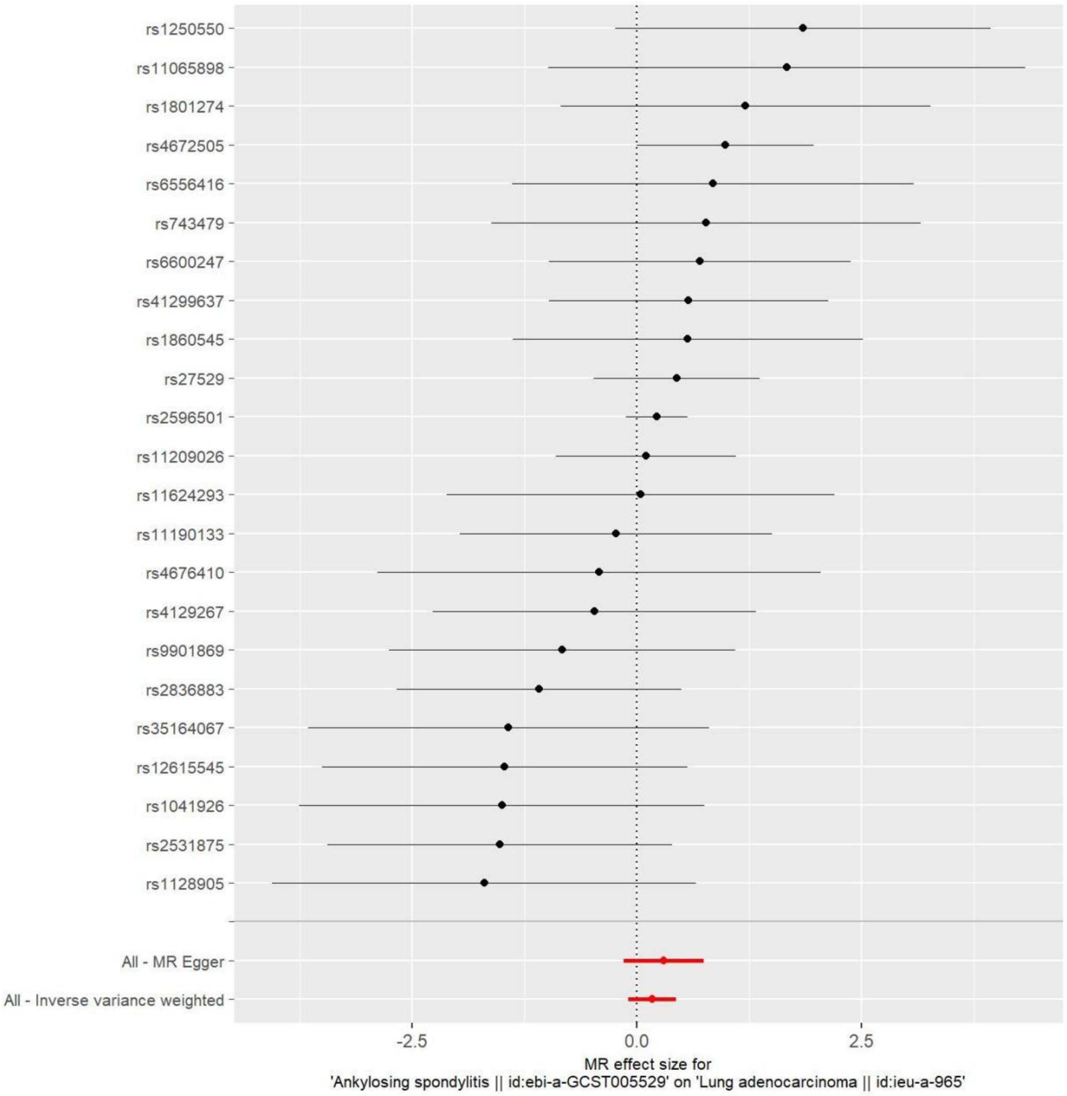
rs1250550
rs11065898
rs1801274
rs4672505
rs6556416
rs743479
rs6600247
rs41299637
rs1860545
rs27529
rs2596501
rs11209026
rs11624293
rs11190133
rs4676410
rs4129267
rs9901869
rs2836883
rs35164067
rs12615545
rs1041926
rs2531875
rs1128905
All - MR Egger
All - Inverse variance weighted
-2.5
0.0
2.5
MR effect size for
'Ankylosing spondylitis || id:ebi-a-GCST005529' on 'Lung adenocarcinoma || id:ieu-a-965'

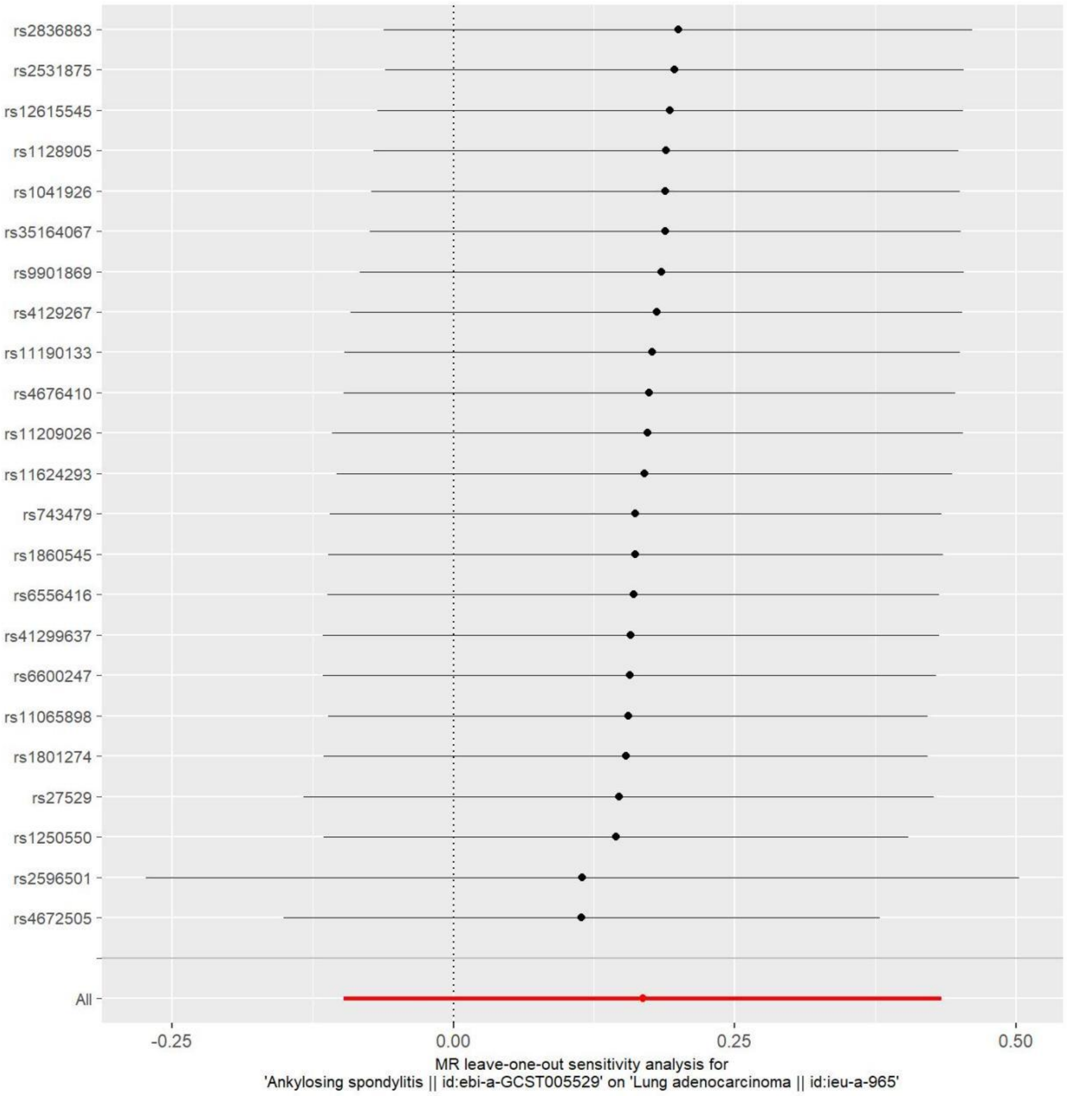
rs2836883
rs2531875
rs12615545
rs1128905
rs1041926
rs35164067
rs9901869
rs4129267
rs11190133
rs4676410
rs11209026
rs11624293
rs743479
rs1860545
rs6556416
rs41299637
rs6600247
rs11065898
rs1801274
rs27529
rs1250550
rs2596501
rs4672505
All
-0.25
0.00
0.25
0.50
MR leave-one-out sensitivity analysis for
'Ankylosing spondylitis || id:ebi-a-GCST005529' on 'Lung adenocarcinoma || id:ieu-a-965'

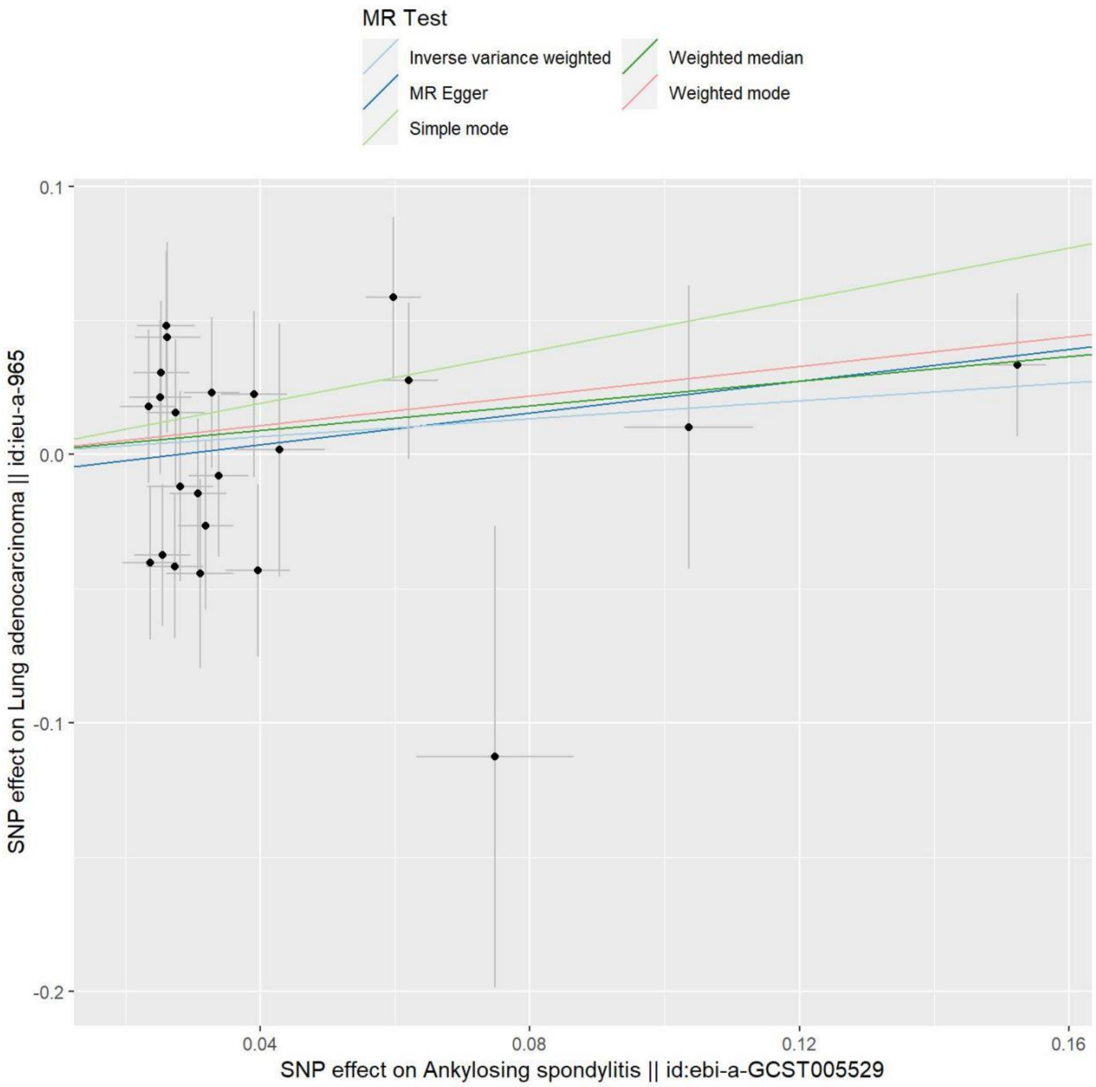
MR Test
Inverse variance weighted
MR Egger
Simple mode
Weighted median
Weighted mode
0.1
0.0
-0.1
-0.2
0.04
0.08
0.12
0.16
SNP effect on Lung adenocarcinoma || id:ieu-a-965
SNP effect on Ankylosing spondylitis || id:ebi-a-GCST005529

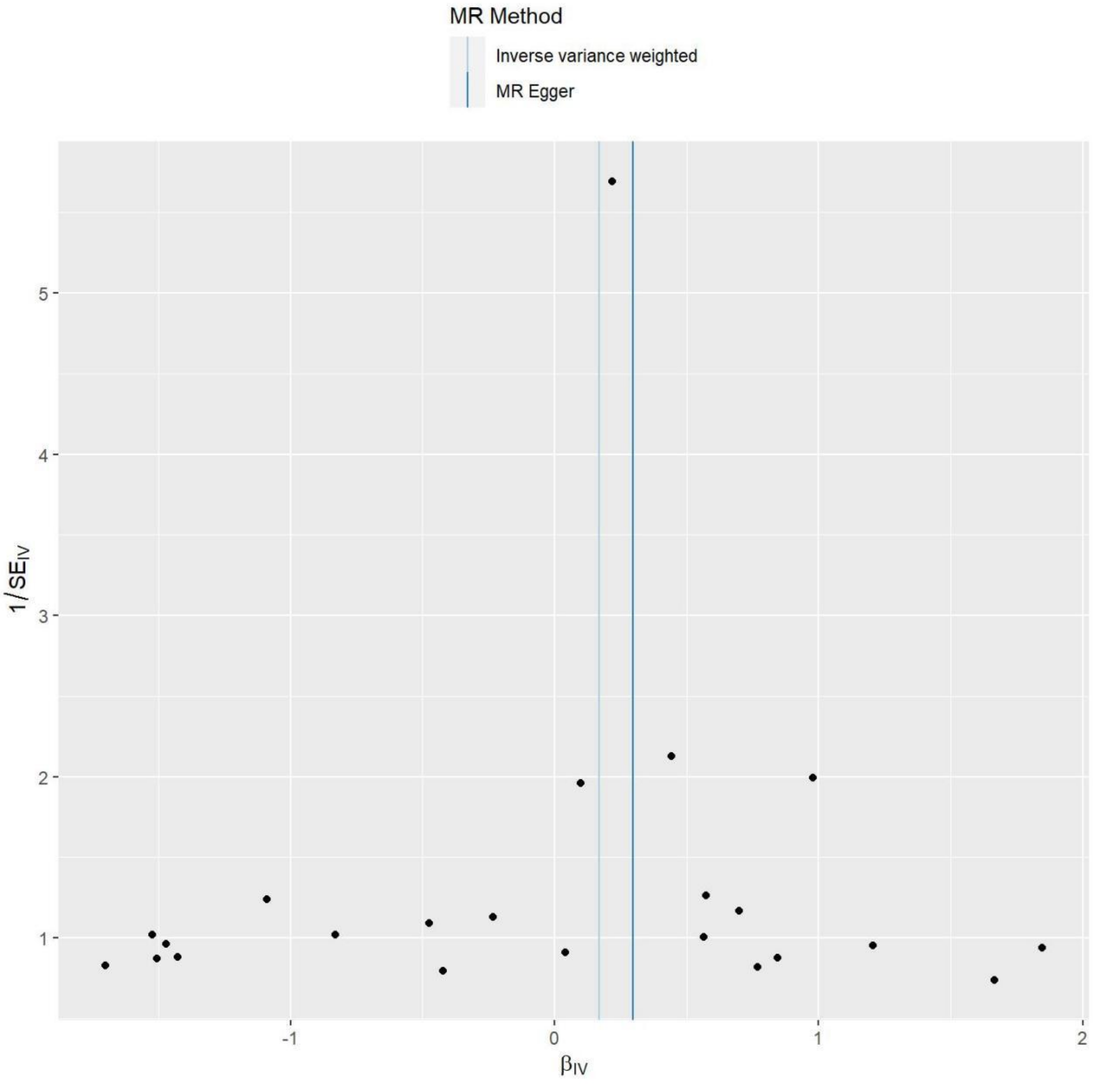
MR Method
Inverse variance weighted
MR Egger
1/SE$_{IV}$
5
4
3
2
1
-1
0
1
2
β$_{IV}$

Supplementary Figure 4. MR effect size, leave-one-out analysis, MR-Egger scatter plot, and funnel plot for AS on lung cancer (dataset ID: ieu-a-966).

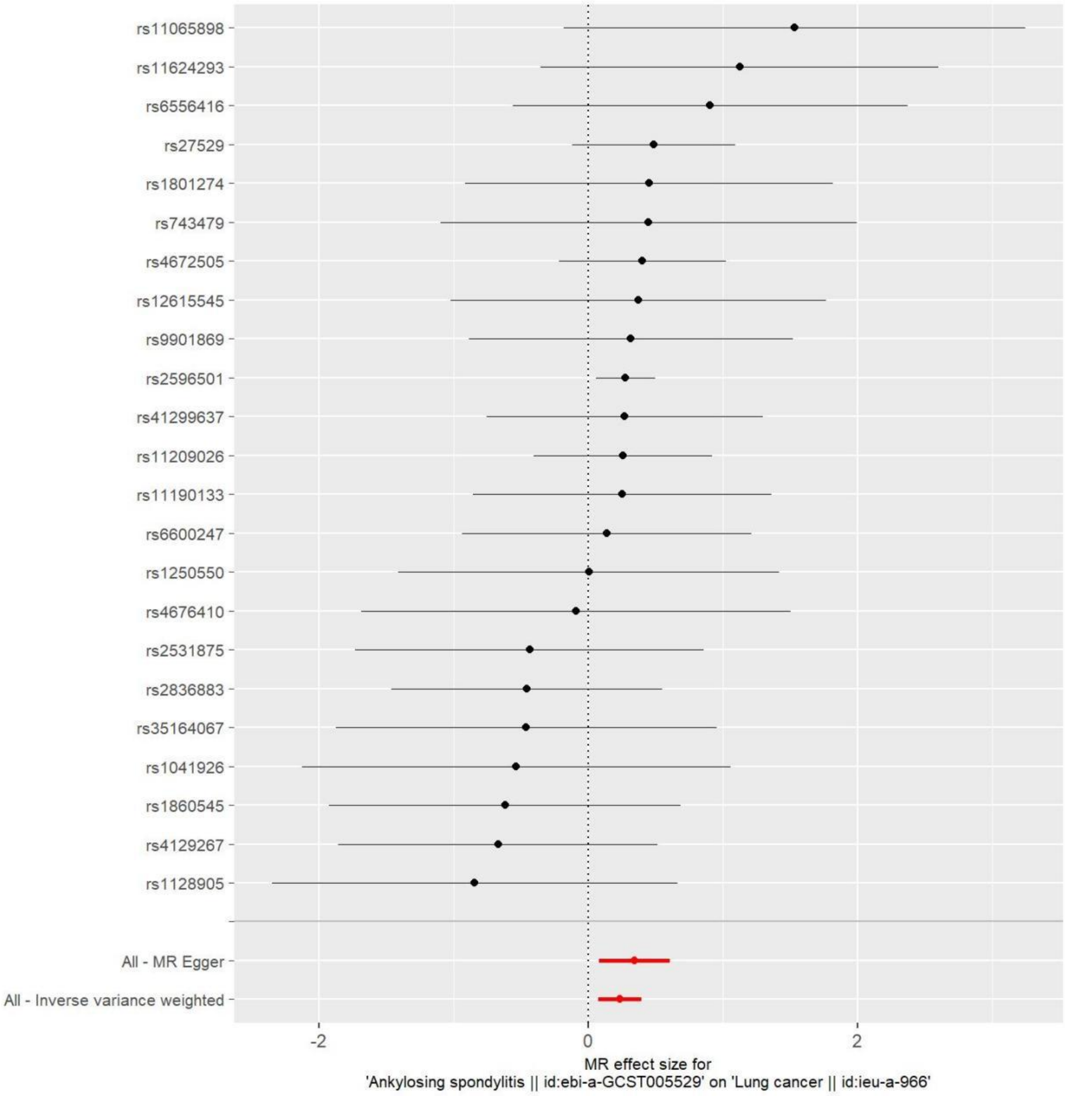

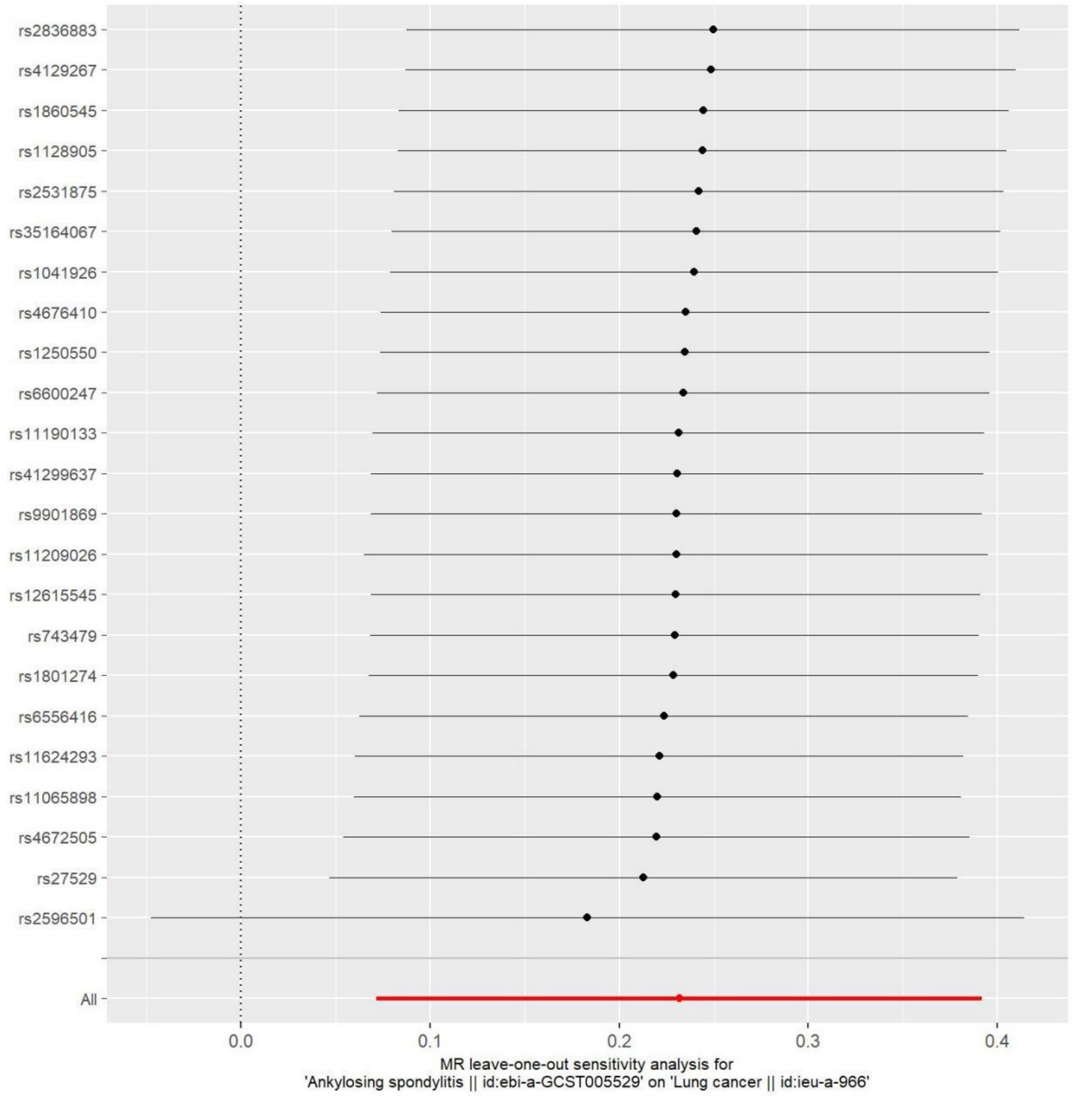
rs2836883
rs4129267
rs1860545
rs1128905
rs2531875
rs35164067
rs1041926
rs4676410
rs1250550
rs6600247
rs11190133
rs41299637
rs9901869
rs11209026
rs12615545
rs743479
rs1801274
rs6556416
rs11624293
rs11065898
rs4672505
rs27529
rs2596501
All
0.0
0.1
0.2
0.3
0.4
MR leave-one-out sensitivity analysis for
'Ankylosing spondylitis || id:ebi-a-GCST005529' on 'Lung cancer || id:ieu-a-966'

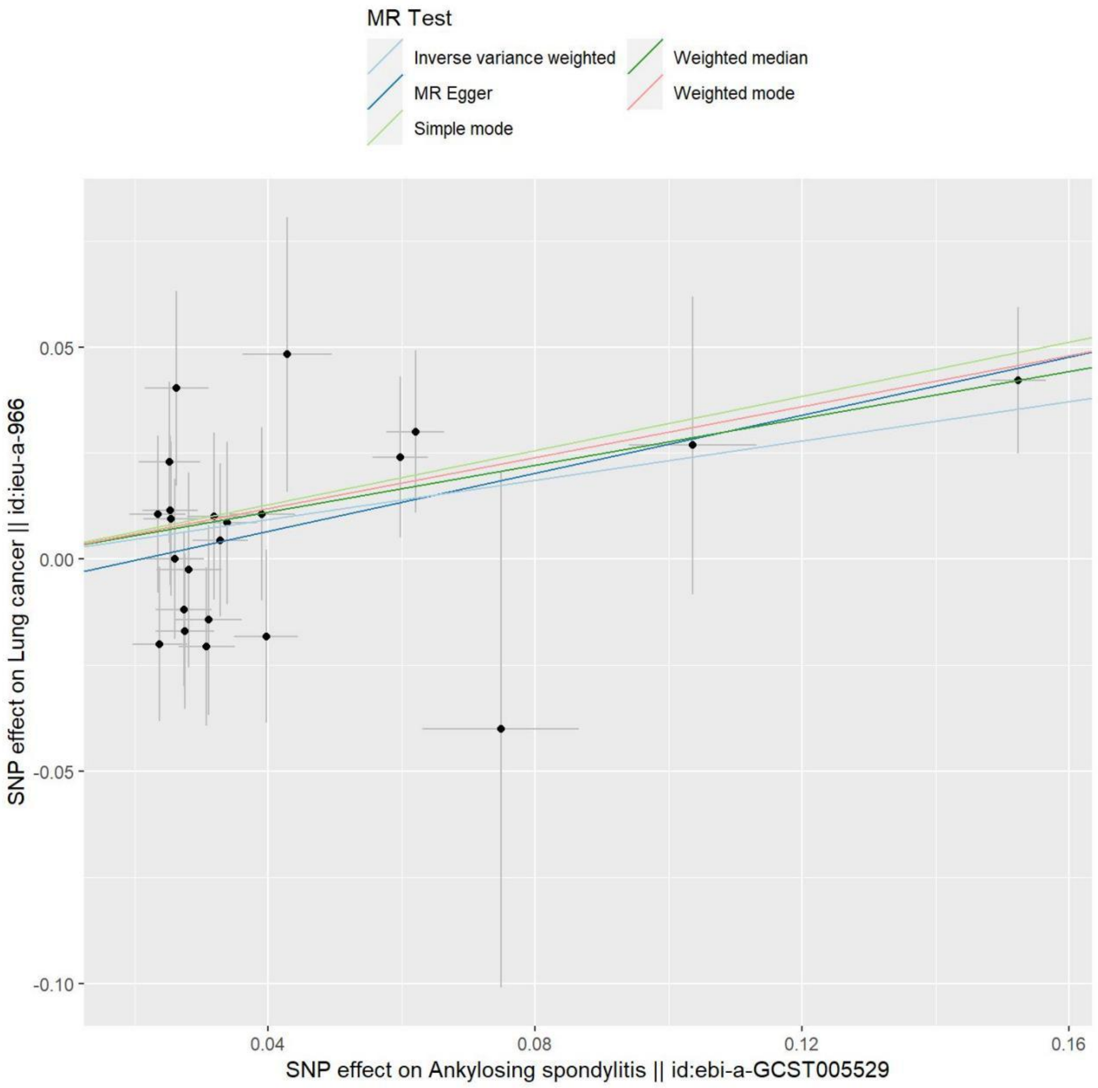
MR Test
Inverse variance weighted
MR Egger
Simple mode
Weighted median
Weighted mode
0.05
0.00
-0.05
-0.10
0.04
0.08
0.12
0.16
SNP effect on Lung cancer || id:ieu-a-966
SNP effect on Ankylosing spondylitis || id:ebi-a-GCST005529

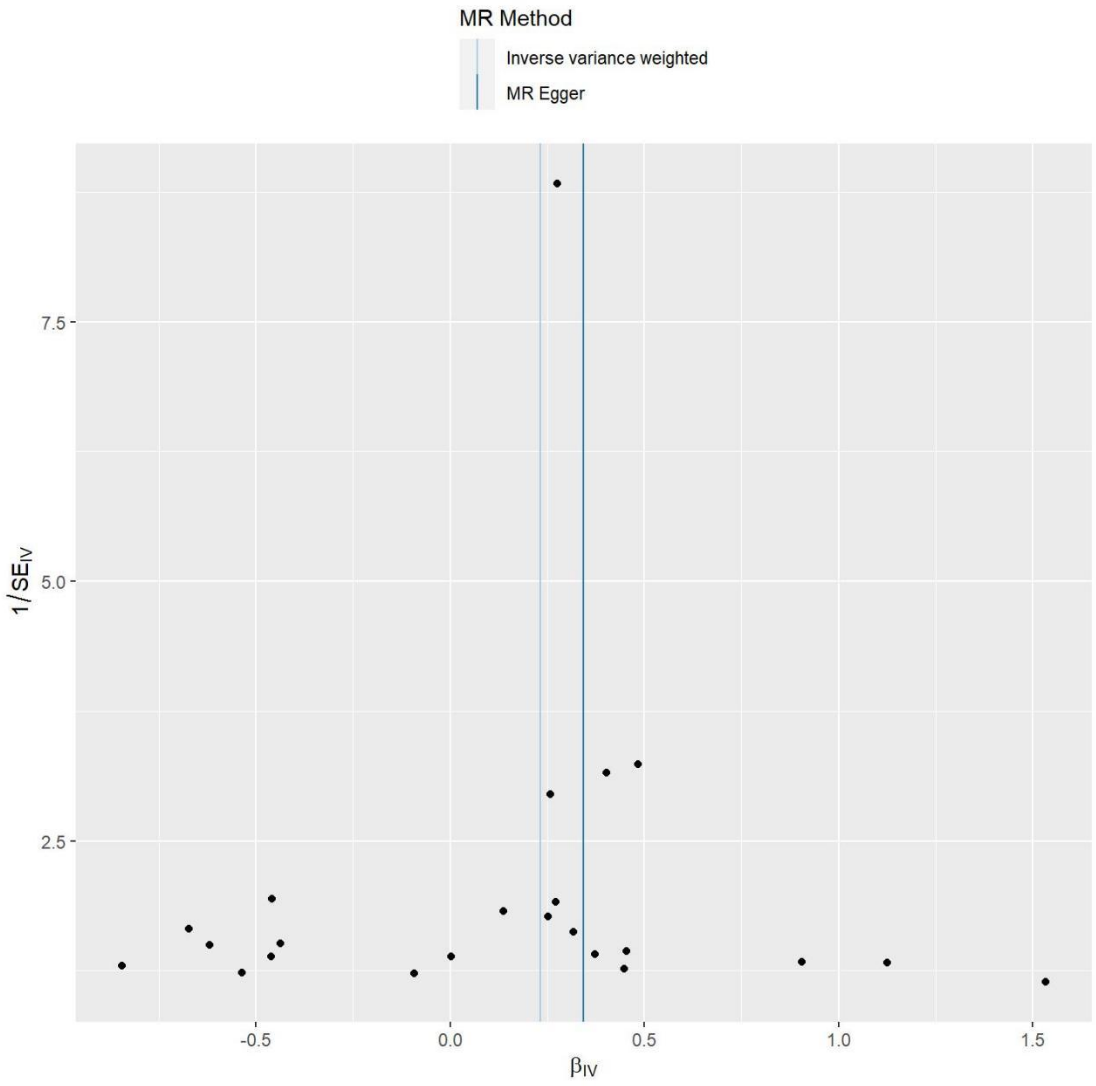
MR Method
Inverse variance weighted
MR Egger
7.5
5.0
2.5
1/SE_IV
-0.5
0.0
0.5
1.0
1.5
β_IV

Supplementary Figure 5. MR effect size, leave-one-out analysis, MR-Egger scatter plot, and funnel plot for AS on squamous cell lung cancer(dataset ID: ieu-a-967).

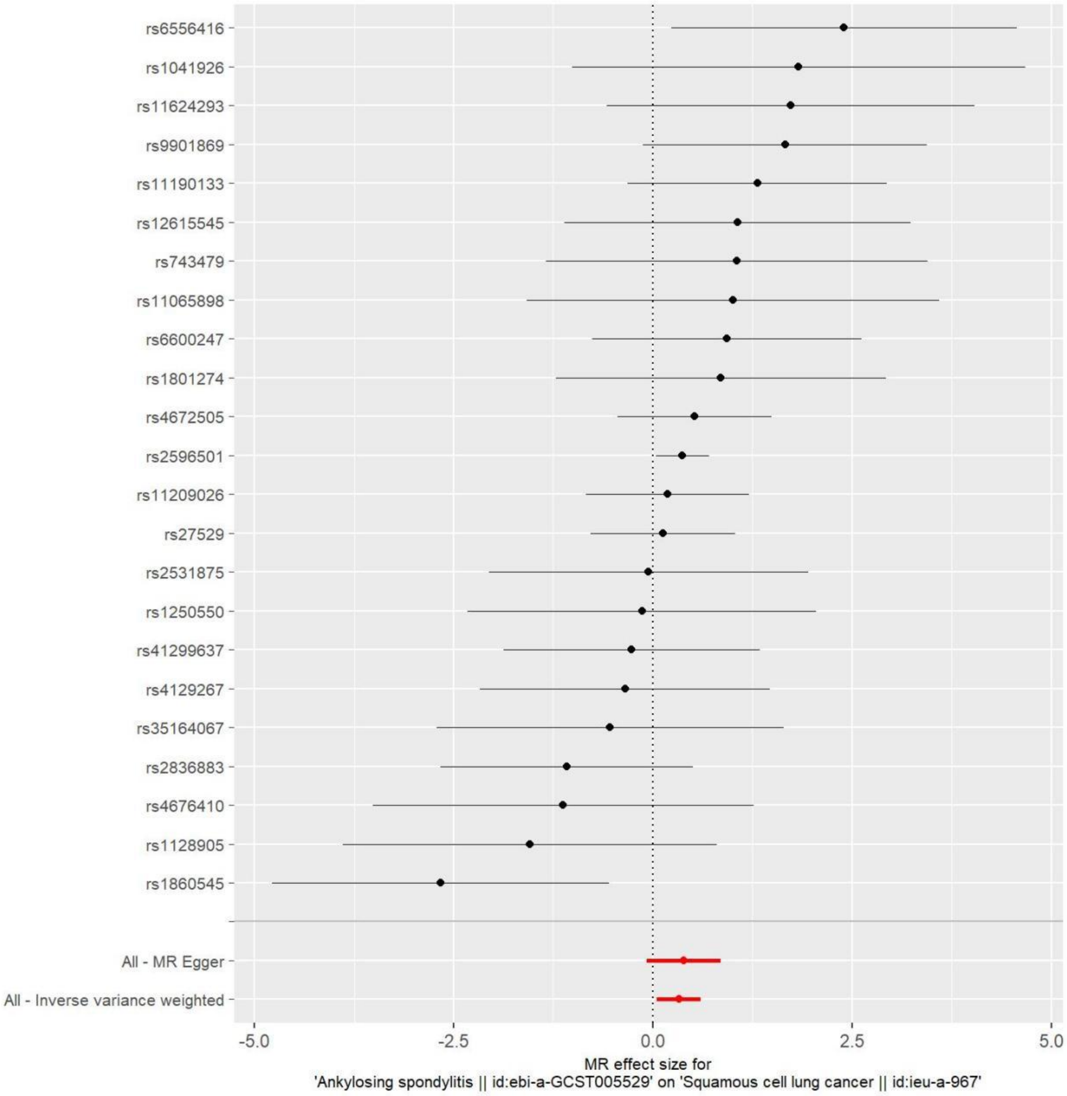

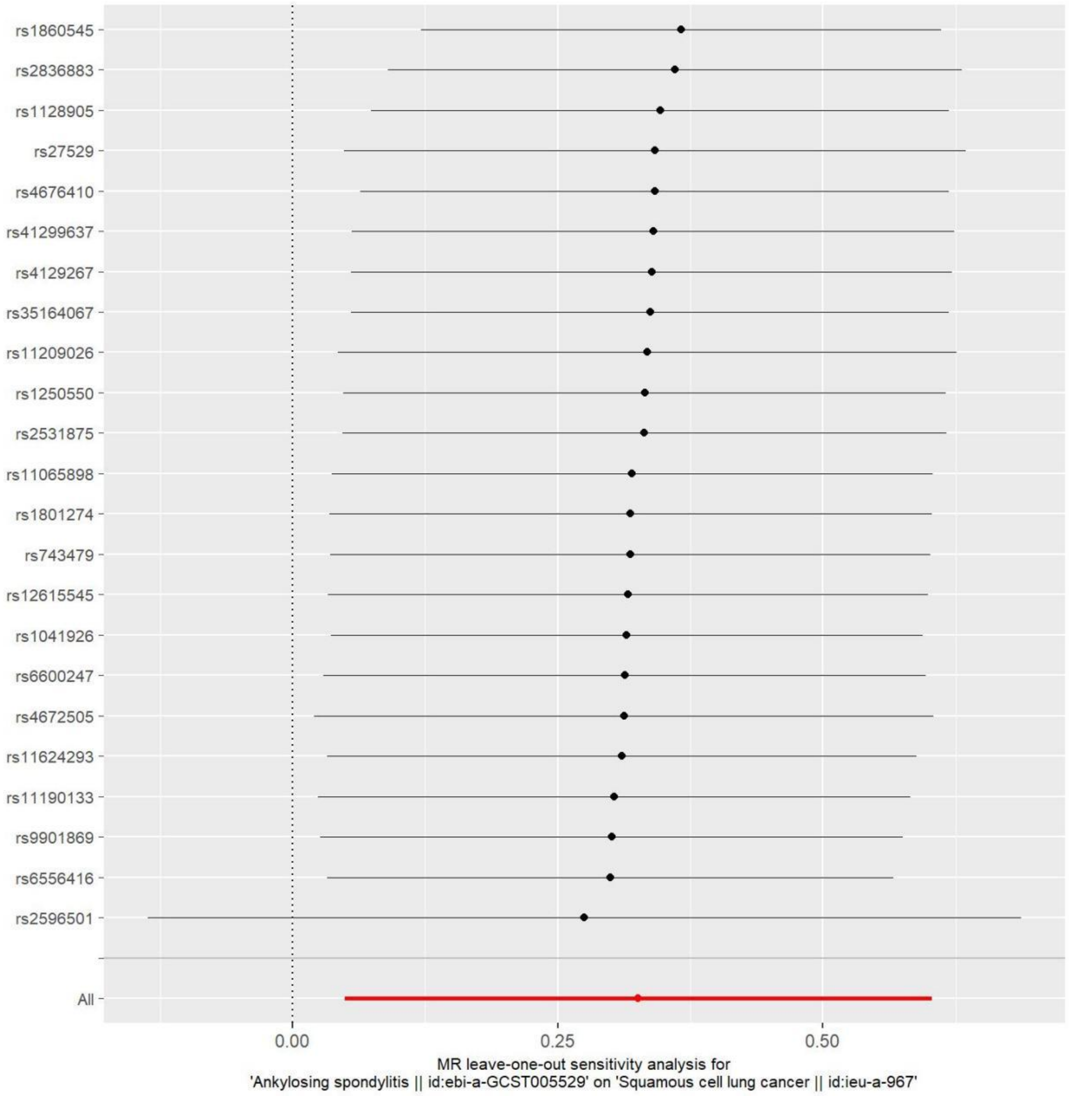
rs1860545
rs2836883
rs1128905
rs27529
rs4676410
rs41299637
rs4129267
rs35164067
rs11209026
rs1250550
rs2531875
rs11065898
rs1801274
rs743479
rs12615545
rs1041926
rs6600247
rs4672505
rs11624293
rs11190133
rs9901869
rs6556416
rs2596501
All
0.00
0.25
0.50
MR leave-one-out sensitivity analysis for
'Ankylosing spondylitis || id:ebi-a-GCST005529' on 'Squamous cell lung cancer || id:ieu-a-967'

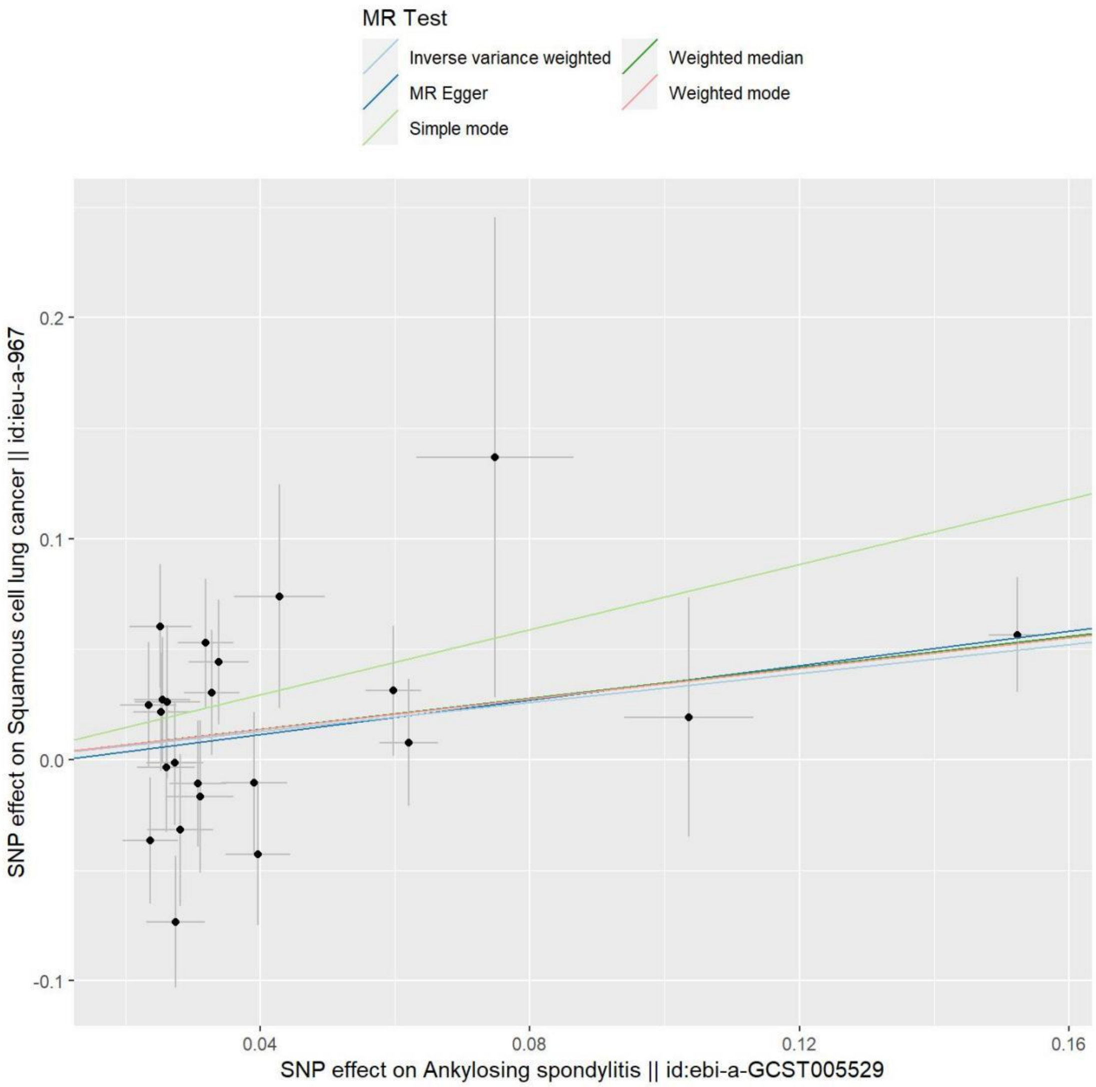
MR Test
Inverse variance weighted
MR Egger
Simple mode
Weighted median
Weighted mode
0.2
0.1
0.0
-0.1
0.04
0.08
0.12
0.16
SNP effect on Squamous cell lung cancer || id:ieu-a-967
SNP effect on Ankylosing spondylitis || id:ebi-a-GCST005529

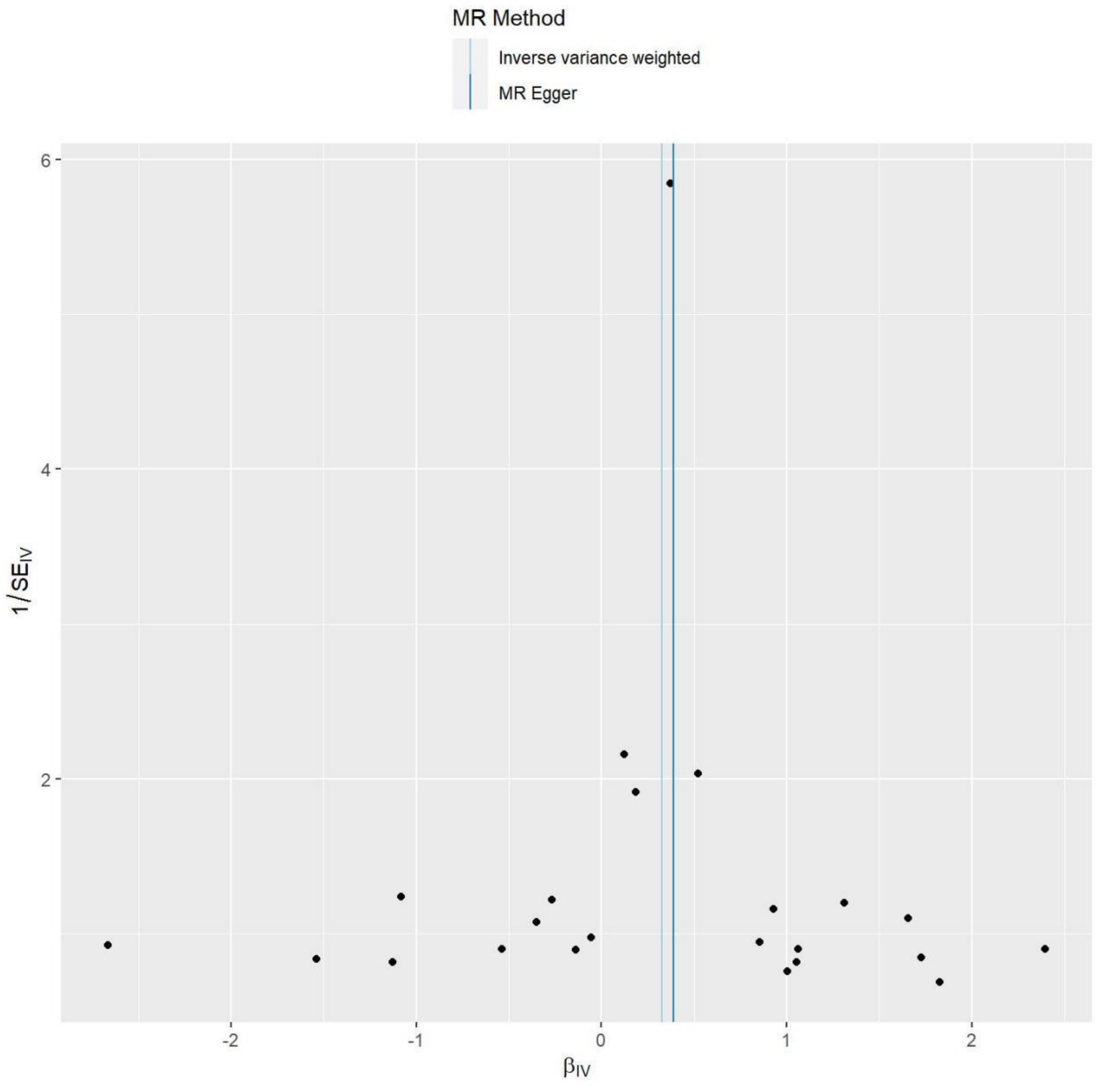
MR Method
Inverse variance weighted
MR Egger
1/SE_IV
β_IV
6
4
2
-2
-1
0
1
2

Supplementary Figure 6. MR effect size, leave-one-out analysis, MR-Egger scatter plot, and funnel plot for AS on lung cancer (dataset ID: ieu-b-4954).

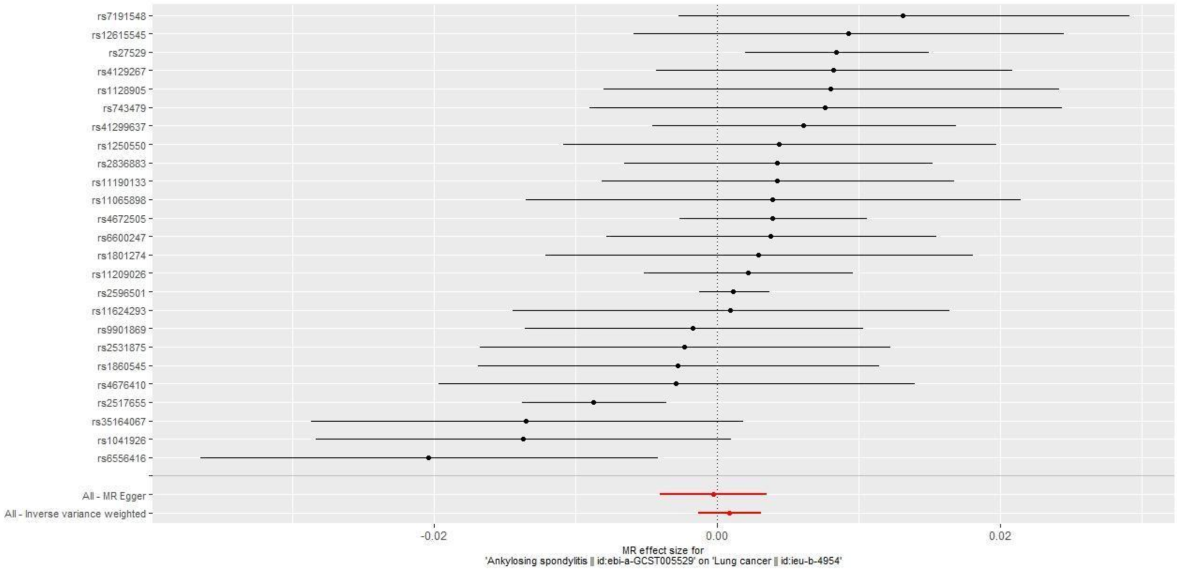

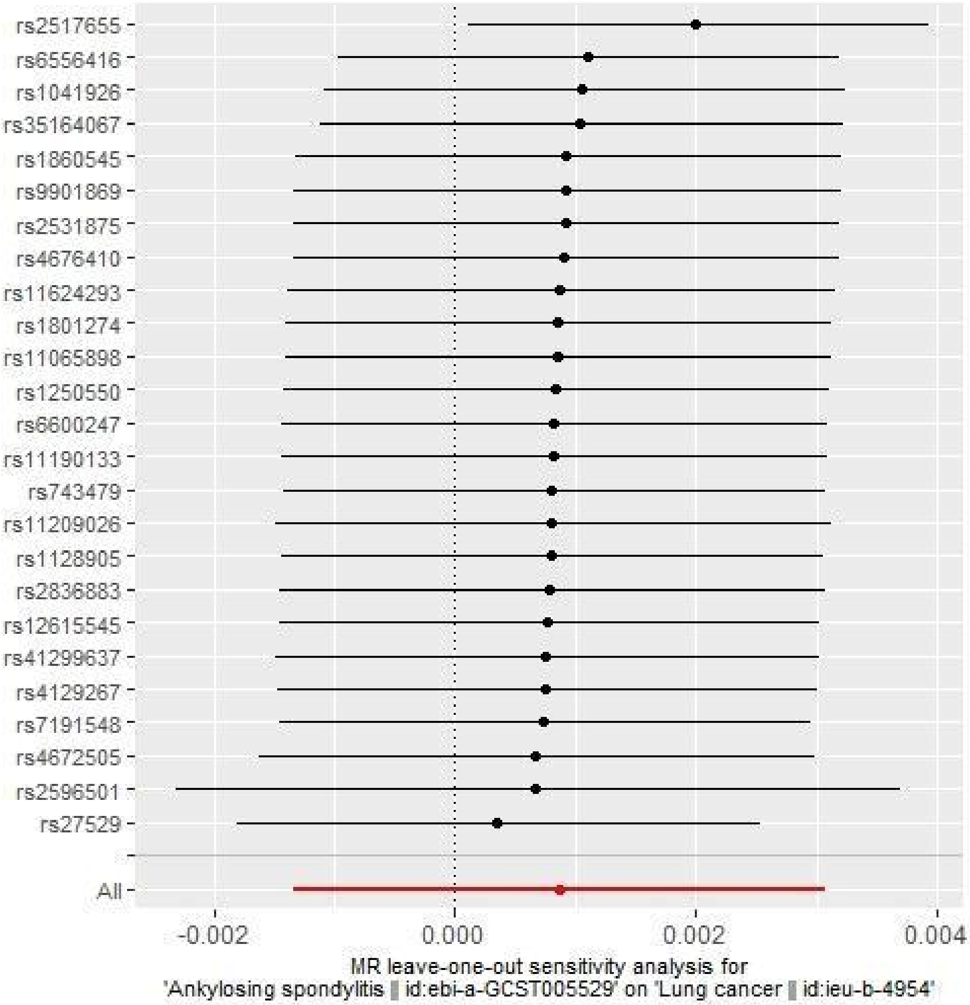

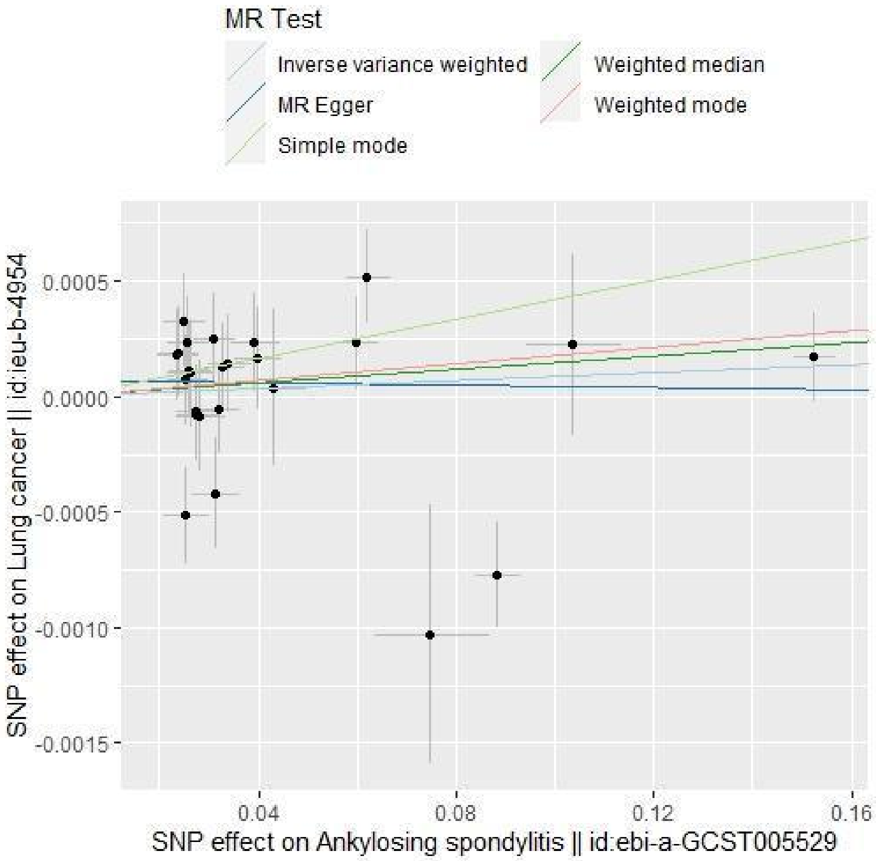
MR Test
Inverse variance weighted
MR Egger
Simple mode
Weighted median
Weighted mode
0.0005
0.0000
-0.0005
-0.0010
-0.0015
SNP effect on Lung cancer || id:ieu-b-4954
0.04
0.08
0.12
0.16
SNP effect on Ankylosing spondylitis || id:ebi-a-GCST005529

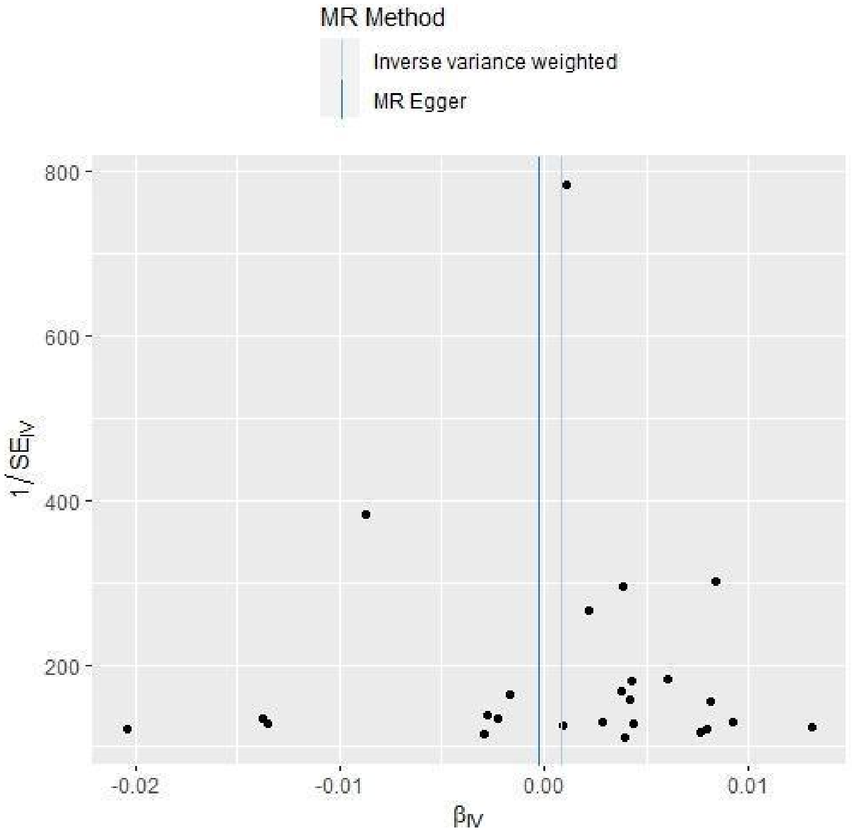
MR Method
Inverse variance weighted
MR Egger
800
600
400
200
1/SEIV
-0.02
-0.01
0.00
0.01
βIV

Supplementary Figure 7. MR effect size, leave-one-out analysis, MR-Egger scatter plot, and funnel plot for AS on Cancer code self-reported: lung cancer (dataset ID: ukb-a-54).

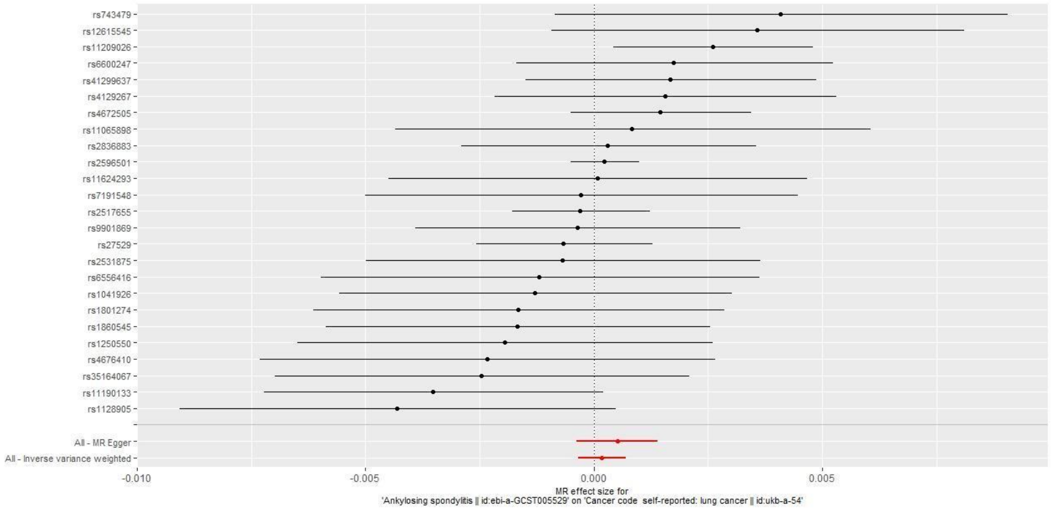

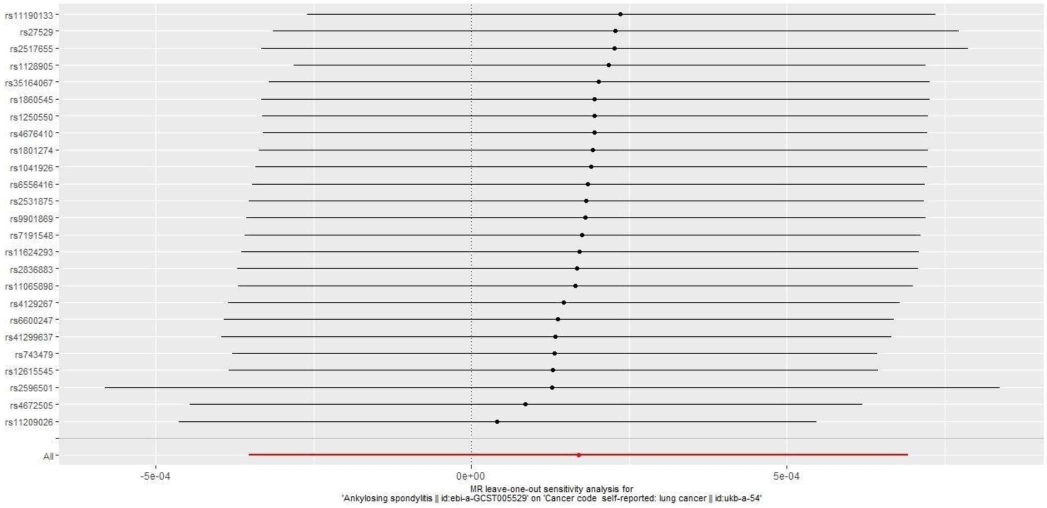

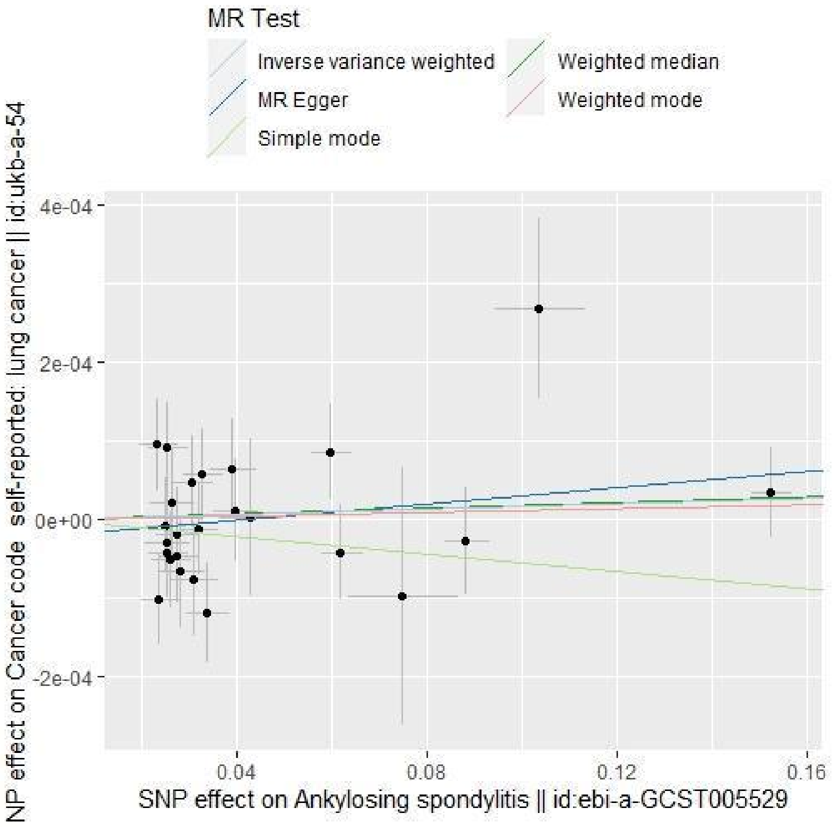

MR Test
Inverse variance weighted
MR Egger
Simple mode
Weighted median
Weighted mode
4e-04
2e-04
0e+00
-2e-04
0.04
0.08
0.12
0.16
SNP effect on Ankylosing spondylitis || id:ebi-a-GCST005529
SNP effect on Cancer code self-reported: lung cancer || id:ukb-a-54

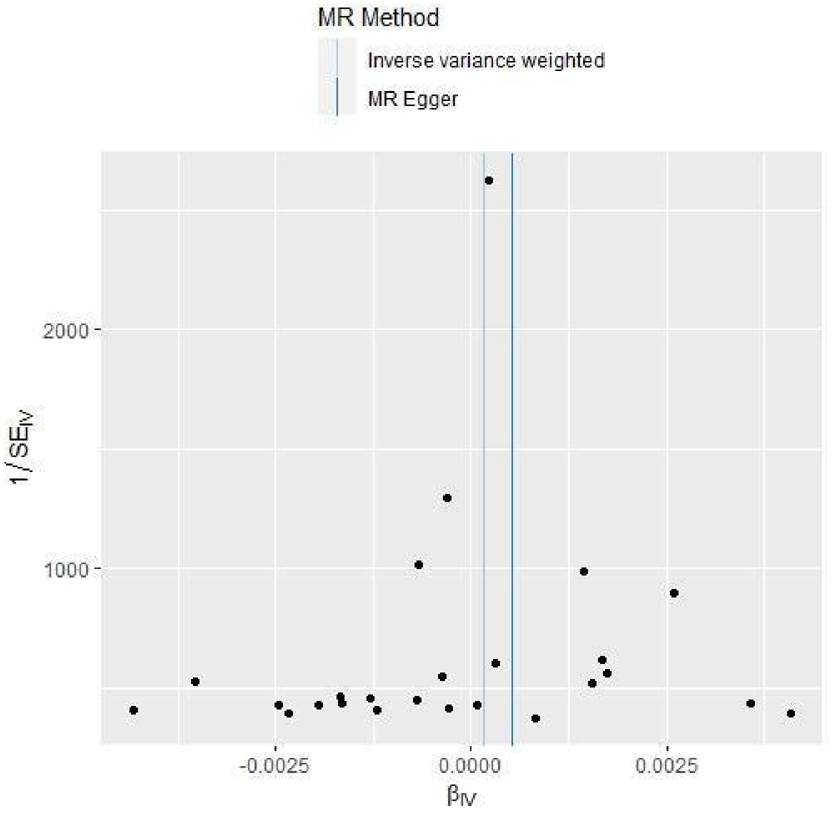

MR Method
Inverse variance weighted
MR Egger
2000
1000
-0.0025
0.0000
0.0025
$1/SE_{IV}$
β_{IV}
